# Wildlife density estimation by distance sampling: A novel technique with movement compensation

**DOI:** 10.1371/journal.pone.0310020

**Published:** 2024-10-16

**Authors:** David G. Morgan, John R. Gibbens, Ed T. Conway, Graham Hepworth, James Clough

**Affiliations:** 1 School of Biosciences, University of Melbourne, Parkville, Australia; 2 School of Mathematics and Statistics, University of Melbourne, Parkville, Australia; University of Glasgow College of Medical Veterinary and Life Sciences, UNITED KINGDOM OF GREAT BRITAIN AND NORTHERN IRELAND

## Abstract

Estimates of population density are fundamental to wildlife conservation and management. Distance sampling from line transects is a widely used sample count method and is most often analysed using *Distance* software. However, this method has limited capabilities with mobile populations (e.g., birds), which tend to encounter an observer more often than immobile ones. This paper presents a novel distance sampling method based on a different set of models and assumptions, named *WildlifeDensity* after its associated software. It is based on mechanistic modelling of visual detections of individuals or groups according to radial distance from the observer or perpendicular distance from the transect line. It also compensates for population–observer relative movement to avoid the detection overestimates associated with highly mobile populations. The models are introduced in detail and then tested in three ways: 1) *WildlifeDensity* is applied to several ‘benchmark’ populations of known density and no-to-low mobility, 2) the movement compensation model is tested on two highly mobile songbird populations, and 3) a fairly difficult case is analysed: a low-density, highly mobile bird population in a forest habitat. The results show that 1) using either radial or perpendicular distance data from surveys of immobile populations, *WildlifeDensity* provides similar estimates (and errors) to *Distance*, with radial *WildlifeDensity* analysis appearing to be slightly better for surveys of low-mobility populations (kangaroos), 2) the movement compensation model effectively removes the correlation between observer speed and detection numbers, and 3) *WildlifeDensit*y provides acceptable estimates where conventional *Distance* analysis overestimates density due to high movement. In summary, *WildlifeDensity* extends the capabilities of distance sampling by 1) compensating for movement, 2) not requiring complete detectability on the transect line and 3) supporting the use of radial distances, which simplifies fieldwork and increases measurement accuracy.

## Introduction

Reliable estimates of terrestrial wildlife numbers are in continual demand, particularly for populations of conservation interest or that are overabundant. Such estimates are commonly obtained through total counts, sample counts or mark-recapture estimates [1].

In line transect distance sampling, observers travel along transects and scan-search the surrounding habitat to count an animal population [2]. Commonly, multiple transects are set according to a random or systematic sampling design. Habitats may vary in structure, composition and observing conditions, affecting detectability. Animals are counted upon detection and their radial distances *r* from the observer measured. The lateral angle *θ* between transect and target bearings is often measured, allowing computation of the ‘perpendicular’ distance from transect line to target, *y* = *r*sin*θ*. Survey data (e.g., counts, distances, angles, transect lengths) are entered into software such as *Distance* [3], which then estimates population density and variance.

**Fig 1:**
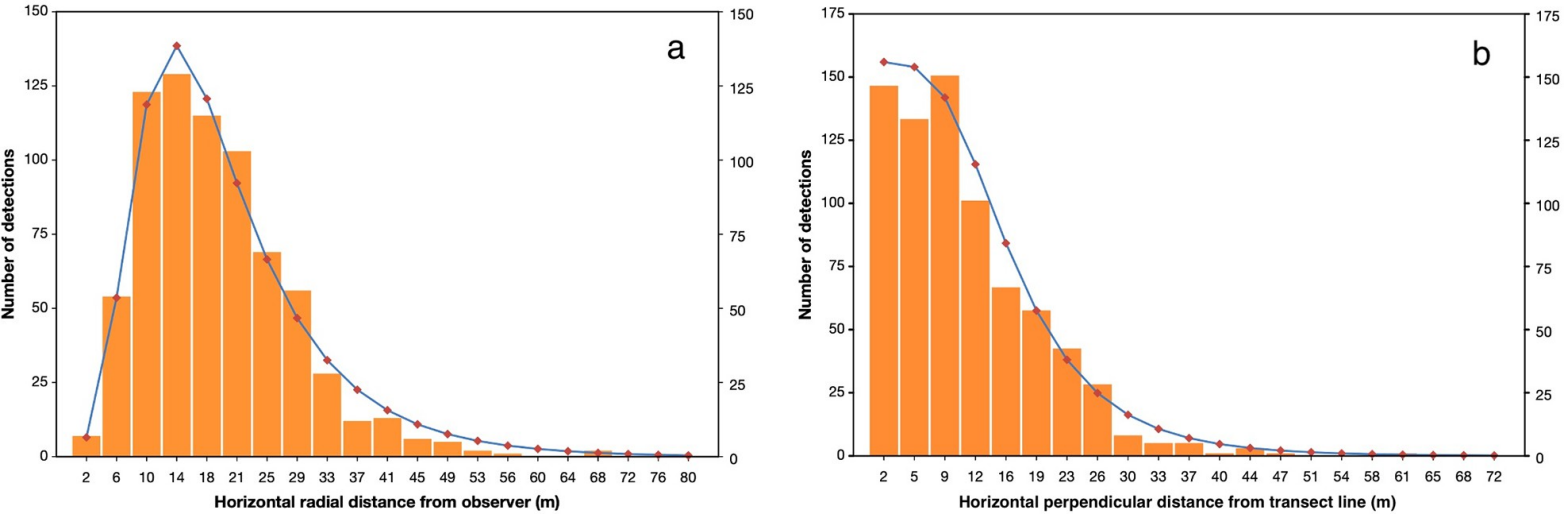
Typical frequency distributions of (a) radial and (b) perpendicular distance data. The data are from the same walked line transect survey of grey fantail songbirds and are fitted with *WildlifeDensity* models (lines).

When plotted, population detection data display distinctive frequency distributions (Fig 1). Radial distance distributions are typically bell-shaped and right-skewed; perpendicular distributions are commonly reversed S-curves. Their consistency allows detection-distance distributions to be modelled and used to estimate population density. Over the years, several such estimators have been developed and used with varying degrees of success [4].

Since the 1980s, *Distance* software has provided several density estimators, of which the ‘conventional’ estimator is probably the most commonly used [5]. *Distance* uses perpendicular distance data, as radial data can cause bias [6]. The accuracy and precision of its density estimates have proven satisfactory in surveys of terrestrial herbivores (e.g., deer, kangaroos) provided sample sizes are adequate and the modelling assumptions are met [7, 8]. The process involves 1) data collection and 2) analysis using software.

### *Distance* assumptions

Line transect distance sampling methods are generally based on assumptions that animals differ in their detectability to an observer due to differences in their conspicuousness (appearance and behaviour), habitat (vegetation, topography and weather between the observer and animals) and observer searching methods (protocol, skill). Detectability typically declines with distance from an observer.

For ‘conventional’ *Distance* surveys based on perpendicular distances of animals from the transect line, the following key assumptions apply [2, 9]:

1. *Location independence*. Surveyed populations are distributed independently of transect lines.
2. *Detection certainty*. Animals at the transect line are detected with certainty (i.e., none are missed).
3. *Measurement accuracy*. Detection distances, angles and counts are accurately determined.
4. *Relative immobility*. a) Animals do not move in response to approaching observers before they are detected [10], and b) the observer’s overall rate of travel is faster than the independent (non-responsive) movement of the population.

These key assumptions need to be met for density estimates by *Distance* to be reliable. If an assumption is likely to be violated, there may be a valid alternative method (e.g., [11] if detection on the transect line is uncertain). Further assumptions include: animals are counted only once unless they independently move out of and then back into the field of view following detection; consistent, compatible search methods are used within a survey; and an adequate sample size is obtained, which is recommended to comprise at least 60–80 observations [12].

### Data analysis

Perpendicular distance data typically have a frequency distribution similar in shape to Fig 1(B). If Assumption 2 of *Distance* is met, detectability should be complete (detection probability *p* = 1) on the transect line and decline with distance from it. The actual relationship is unknown, so *Distance* fits mathematical functions (e.g., half-normal) to the frequency distribution, using adjustment terms to improve fitting [4]. The best-fitting function is treated as the detection function *g*(*x*) of the data [3].

The total number of individuals *n* within a strip of length *L* and width 2*w* centred on the transect line is also estimated (*w* being the *effective half-width*). Function *g*(*x*) is used to estimate the true number of animals within *w* by estimating how many are undetected. The program estimates density as *D = n*/2*wL* and calculates its coefficient of variation.

### Possible limitations

The main assumptions of *Distance*, especially complete detectability on the transect line, can be unmet without the investigator realizing it, resulting in underestimated density [10]. Animals that change location more rapidly than the observer (e.g., flying birds) are at risk of being detected more than once, leading to overestimated density [13]. This is because they have the potential to leave and re-enter the visual field multiple times, whereas stationary animals can only pass through the visual field once (as the observer passes by). Errors can be made in counting and measuring distances and angles [9]. Inconsistent search procedures can complicate comparisons between surveys.

### A novel method

This paper describes a novel distance sampling approach and associated software called *WildlifeDensity* [14]. It originated from a 1970s study of dispersion and abundance in mobile, mainly arboreal birds of old-growth Australian forests and woodlands. Arboreal birds are often difficult to survey by distance sampling, as they may 1) be above the observer’s horizontal plane (high in the canopy), 2) exist in low numbers, 3) be continually moving, and 4) be sometimes hidden (e.g., in nests).

*WildlifeDensity* was designed for such situations, employing mechanistic models of the way in which detectability varies with distance in terrestrial habitats. During survey programs for Australian state and federal departments (e.g., [15]), its scope was later broadened to include vertebrates of general terrestrial habitats, with its software developed in tandem. The models attempt to approximate animal conspicuousness (e.g., size, colour, behaviour) and factors that obscure them (e.g., vegetation, topography, weather) while allowing for distinctive population behaviours and habitat attributes.

This paper summarizes *WildlifeDensity*’s assumptions and procedures, outlines its structure and tests its effectiveness as a terrestrial wildlife density estimator.

## Methods and models

### A survey protocol

Observer search procedures affect the detection data collected along transects and the resulting density estimates. We provide a detailed field protocol, as observer search behaviour has an important influence on the results. Furthermore, consistency in search behaviour increases the potential for data obtained at different times and by different observers to be pooled to increase the sample size. The fieldwork protocol set out below was developed and refined in a wide range of surveys in tandem with *WildlifeDensity* software. Its consistent use by all observers involved in a survey is strongly recommended to maximise the likelihood that robust density estimates are obtained.

#### Detections

A *detection* is defined as an individual or small cluster of animals seen at a particular horizontal distance in the area scanned. Use only visual detections (sightings), since auditory ones (calls) are frequently highly variable and, thus, unreliable, with the detection distances difficult to measure [16]. To simplify the scanning task, restrict population surveys to one species at a time. Ensure that animals are unlikely to move in response to the observer’s approach before detection.

#### Beginning and ending transects

Before beginning a transect, observers take note of, then disregard, any animals already visible from the starting point. Record only *new* detections made once the transect has begun. Record animals visible beyond the transect end point.

#### Making observations

Observers are to (a) travel slowly, quietly and as inconspicuously as possible; (b) continually and evenly scan for animals within the 180° field of view ahead (both sides of the transect line, although single-sided 90° surveys are possible. Scanning 360° is theoretically possible, but is much harder to do and has little benefit as animals behind the observer have usually already been detected); (c) pause to record animals as they are detected while aiming to maintain a *steady average speed* throughout the transect; (d) count only animals visible to the unaided eye (use binoculars only to confirm an uncertain detection but not for scanning or counting clusters); (e) individuals within a short distance of one another (e.g., 5–10 m) can be counted as clusters; (f) treat cluster members initially obscured but later sighted (e.g., as a ridge is crested) as a new detection; (g) measure (never estimate) radial detection distances in the *horizontal plane* with a rangefinder (± 1 m) to the individual or the centre of the cluster; (h) for detections above or below eye-level, also measure the elevation angle *∅* with an inclinometer to allow calculation of the direct-line distance *d* = *r*/cos*∅*. If using perpendicular distances, measure lateral angles relative to the transect line (using a compass or protractor); (i) keep track of counted animals and avoid recounting them unless they *autonomously* move out of sight, then back into view; (j) to determine observer speed, record all transect lengths and durations; (k) if traversing transects containing multiple habitats with differing detectability (e.g., forest, grassland), record the habitat type and the duration of travel through it to allow stratified analysis; and (l) record all data immediately, thoroughly and reliably (e.g., with paper and pencil or a dependable digital device). Data collected using the above methods are also suitable for *Distance* analysis so long as lateral angles are recorded and care is taken not to miss detections on the transect line. We note that the *Distance* literature provides relatively brief information on search behaviour [2].

#### Animals that overtake observers

Animals detected behind the observer (i.e., beyond 90° to either side of directly ahead) are not counted unless the observer detects them entering the scanning area from behind it (observers usually have some awareness of areas just beyond 90°). For such cases, record the distance as zero (0) to signify to the *WildlifeDensity* software that this detection was an *overtake*. During later analysis, the total detection frequencies are then increased according to the proportion of overtakes (usually low). With perpendicular distance surveys, record detections *exactly* on the transect line (i.e., lateral angle 0°, perpendicular distance 0 m) as a near-zero distance to avoid confusion with overtakes (e.g., 0.01 m, which has little effect on the results).

#### Stratification

To increase the precision of density estimates, transects can be divided into segments of similar detectability, most commonly according to vegetation cover (“habitat”; or by weather, lighting, sex in sexually dimorphic species, etc.). Observers estimate the length of transect travelled in each stratum by recording the positions or times at which the strata changed (assuming an overall consistent observer speed). The results from each of these stratified samples are then combined (see Section *Using stratification with line transect surveys*).

### *WildlifeDensity* assumptions

*WildlifeDensity* shares some of the assumptions required for *Distance* (Section *Distance assumptions*), being Assumption 1 (location independence) and Assumption 3 (measurement accuracy). However, complete detection on the transect line is not required (Assumption 2). Assumption 4—that there is no movement (change in location) of animals—can be relaxed. While it remains important that there is no *responsive movement* (a response to an observer’s approach before detection), *non-responsive movement* (the animals’ normal behaviour) can be allowed for. This should be more or less at random in the horizontal plane, as opposed to population-wide movements (such as migration). Both techniques assume that media between the observer and animals (vegetation, atmosphere, topography) reduce detectability with distance. The main models underlying *WildlifeDensity* are described below.

### Modelling the study site

**Fig 2:**
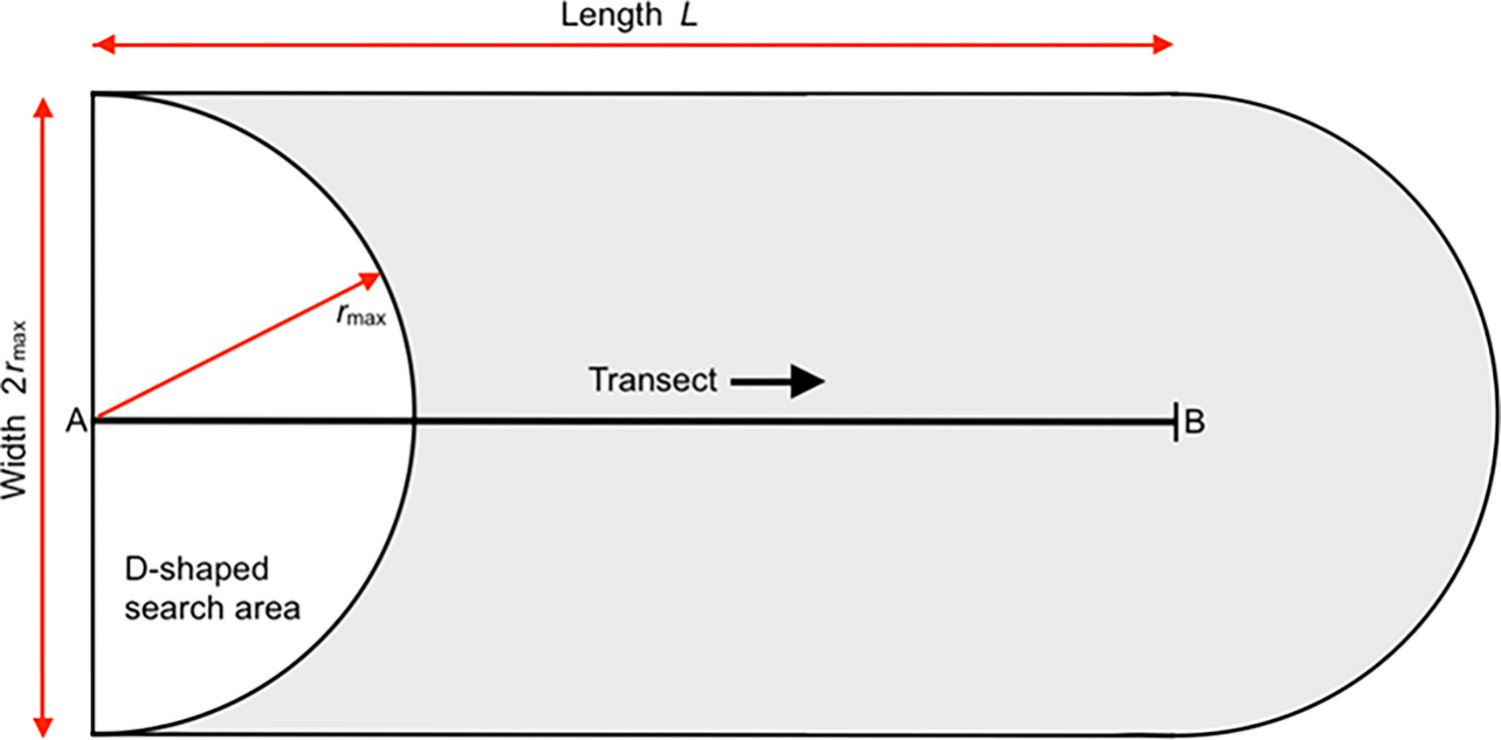
The area passed through by the outermost observing arc (radius = *r*_max_) as an observer travels a transect from A to B (shaded; area = 2*Lr*_max_ m^2^). The diagram is not to scale; in reality, the length will be much greater than the width and any discrepancies at either end (between the transect and search-area start/end points) will be minor.

An observer scanning a horizontal plane ahead to 90° on each side of the transect will have a semi-circular, or D-shaped, search area bounded by an arc (visual detection limit, radius *r*_max_) and a straight line perpendicular to the transect (Fig 2). This area moves forward with the observer; animals enter it and become available for detection when the arc sweeps over them or by overtaking from behind (only possible if they move faster than the observer). Consider a site containing an animal population of density *D*, through which the observer walks a transect of length *L*. As the observer moves forward, the arc passes through an area equal to a rectangle of length *L* and width 2*r*_max_. The population within this rectangle will be *D·*2*Lr*_max_.

**Fig 3:**
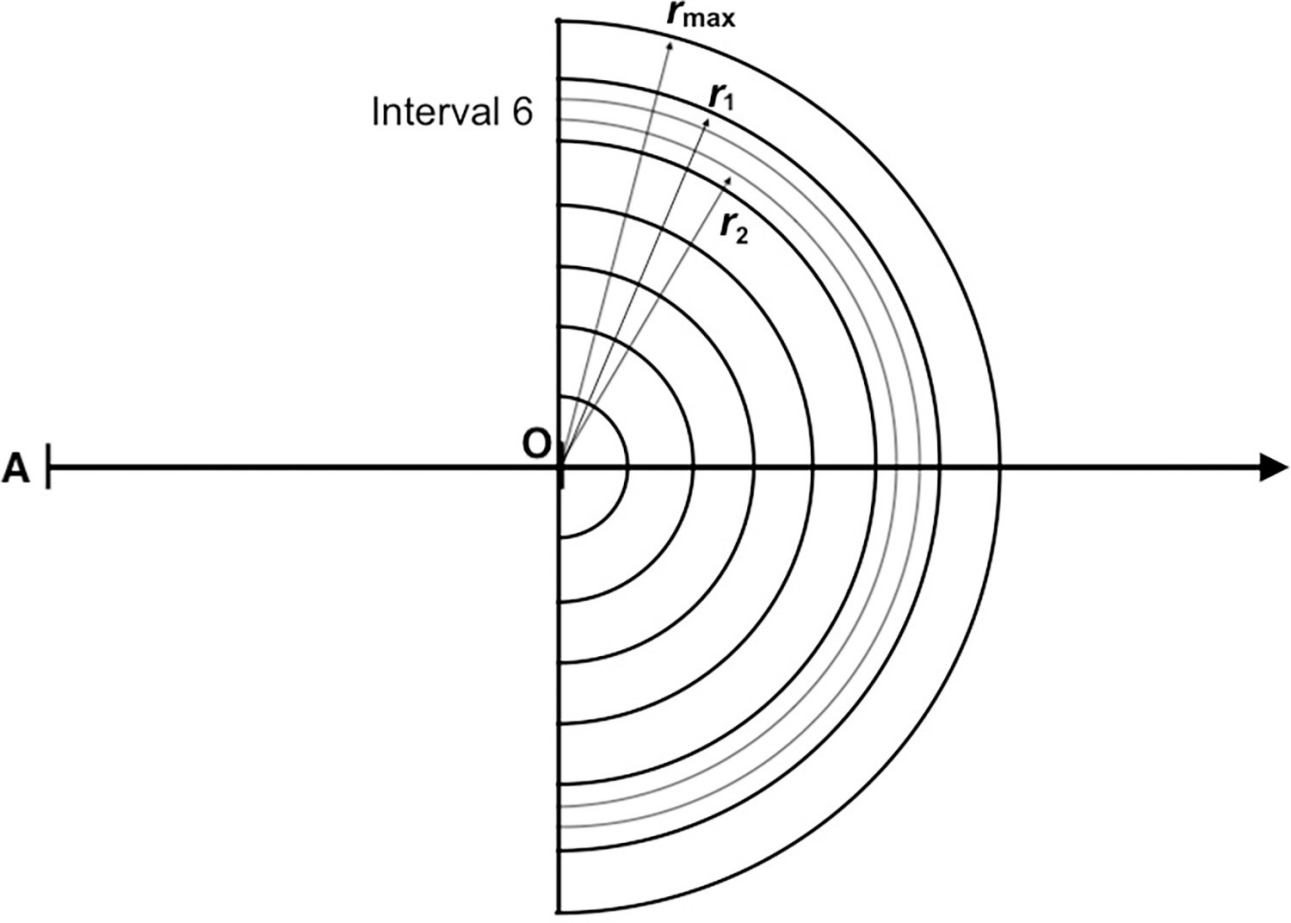
Simplified depiction of observing half-annuli ahead of an observer (O) on a transect line. In this example, there are seven distance intervals, each containing three half-annuli of width Δ*r* (only shown within Interval 6 for simplicity). Detections begin in the most distant half-annulus (bounded by *r*_max_), continue in closer ones (e.g., annuli bounded by *r*_1_ and *r*_2_) and end where the detection probability *p*_d_ = 1, unless some animals remain unseen. The half-annuli pass through areas measuring 2*Lr* (see Fig 2), which vary according to the value of *r*.

If the search area is divided into radial distance intervals, a set of concentric *observing half-annuli* (half-ring-shaped areas) is produced (Fig 3). Imagine a set of, say, *n* ≥ 70 half-annuli, each with a narrow width Δ*r* (where Δ*r* = *r*_max_/*n*); each passes through an area of 2*Lr* (where *r* ranges between *r*_max_ and Δ*r*). In the model, all half-annuli move ahead with the observer in Δ*r*-wide steps. This annulus-by-annulus procedure is fundamental to the *WildlifeDensity* approach and can be applied to radial or perpendicular distance data.

#### Radial distance data

Beginning with the furthest half-annulus (bounded by *r*_max_) and then in nearer ones, animals are detected, recorded and subsequently disregarded as the observer advances. The number of detections is near-zero at *r*_max_ and increases with proximity to the observer up to a certain point; it then declines as the number of animals available for detection decreases. This typically results in a frequency distribution resembling a right-skewed bell curve (Fig 1A). Densities are estimated for the areas passed through by each half-annulus, then combined into distance classes.

#### Perpendicular distance data

**Fig 4:**
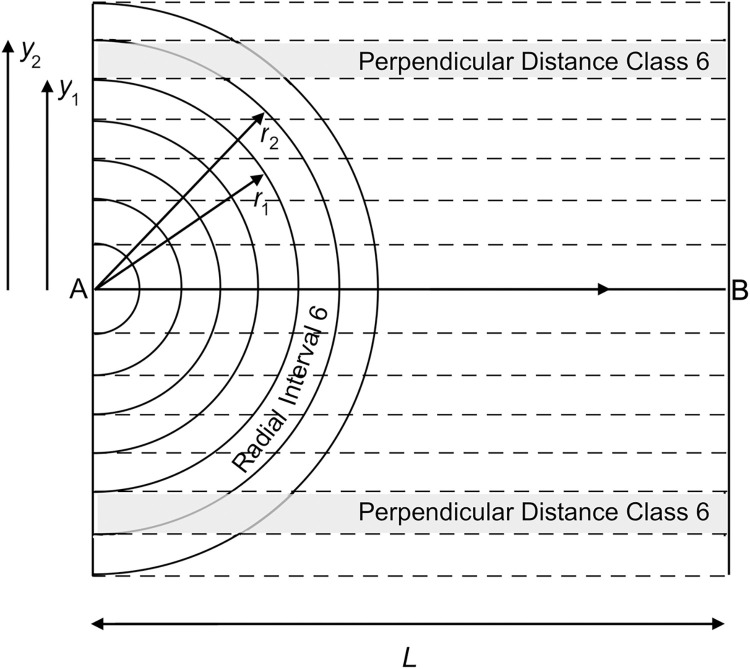
Areas swept out by an interval of observing annuli on transect AB when using perpendicular distances *y*. As with Fig 3, there are seven radial distance intervals, each containing three Δ*r*-wide annuli (not shown). Consider the end of Radial Interval 6 (between *y*_2_ and *y*_1_), which passes through rectangular strips on either side of the transect (shaded; area = Σ*L*Δ*r* m^2^). Each strip is also swept by portions of annuli with greater radii (in this case, Radial Interval 7). Densities are estimated for each perpendicular distance class in sequence from the greatest to least.

A comparable model applies when using perpendicular distances, although the geometry of the sampling situation differs. Here, the program predicts detection numbers within narrow strips parallel to the transect (Fig 4). Densities are estimated for each perpendicular distance class in sequence from the greatest to least. The detection frequency distribution typically has the reversed S-shape of Fig 1B.

### The probability of detection

To see how the models work, consider now a radial distance survey of a population of density *D* dispersed amongst relatively uniform vegetation. Let the transects be *L* m long, the observer and population movement rates known, and *N* be the total number of detections. The task is to estimate the numbers of detections within each half-annulus of radial distance *r* and corresponding direct-line distance *d* (when the observer and population are in the same horizontal plane, *r* = *d*). For this, we need to estimate both the population density *D* at *r* (and *d*) and the probability *P*_*d*_ of detecting an animal at distance *d*.

Our modelling is based on the properties of light and human vision. Detecting an animal requires an observer to visually discriminate it from the background. The probability of detecting an animal by sight at any particular distance—its *visual detectability—*depends mainly on the amount of light reflected from the animal (affecting its *conspicuousness*) and the extent to which it is obscured by atmospheric effects, vegetation and/or topography.

#### Animal conspicuousness

At a very short distance *a*, an animal is always detectable (*p*_*a*_ = 1). Detectability then declines with distance according to the inverse-square law of light transmission. An animal’s probability of being discriminated at *d* can then be represented as *p*_*d*_ *= a*^2^*/d*^2^ (0 *< p*_*d*_ *<* 1). Here, *a* is a constant defined as the *conspicuousness coefficient*, which depends on the species and the habitat it is detected in and is approximately the minimum detection distance (*d*_min_, at which animals will never be undetected). There is also a maximum detection distance *d*_max_, beyond which the animal’s image is too small to discern. Larger animals usually have greater values of *a*, *d*_min_ and *d*_max_ than smaller ones. Movement and background contrast may also affect conspicuousness.

#### Atmospheric attenuation

Image clarity may decline with distance due to atmospheric effects (e.g., mist, fog, rain), reducing detectability in a complex and exponential way representable by *p*_*b*_, where *p*_*b*_ *= e*^−*bd*^ (0 *< p*_*b*_ *<* 1) and *b* is the *atmospheric attenuation coefficient*. In clear weather, except at very long distances (e.g., aerial surveys at sea), *b* will be negligible and can be disregarded. Observers should avoid poor atmospheric conditions (e.g., heavy rain and fog, where *b* is not negligible).

#### Lateral cover

Visual detection also requires a clear line-of-sight, which may be blocked by vegetation and/or topographical cover, as follows. Let *cover* (*c*) represent the total length of solid matter (e.g., tree trunks, foliage; in m) potentially interrupting the line-of-sight between observer and animal. Then, the *mean lateral vegetation cover* (*c*_v_) of a given habitat is the mean proportion of vegetation intercepting all lines-of-sight, for which ∑*d* is the overall line length: *c*_v_ *=* ∑*c*/∑*d*. Cover decreases detectability in proportion to *c*_v_ and is cumulative. Because detectability depends on *unobscured* visibility, if the lateral cover proportion is *c*_v_, the proportion of lines-of-sight unobscured becomes (1 − *c*_v_). The *probability of visibility through lateral cover* (*p*_v_) at *d* can then be modelled as *p*_v_ *=* (1 − *c*_v_)^*d*^.

#### Topographical cover

If the terrain is level, or detection distances short, topography has little effect on detection rate; however, features such as hills may obscure animals. Consider a representative *obscuring distance* (*d*_T_) to a hilltop ahead (e.g., 200 m). Up to *d*_T_, no animal is hidden by topography, while beyond *d*_T_, animals are increasingly obscured up to *d*_max_. In general, the *probability of being unobscured by topography* (*p*_T_) at distances less than *d*_T_ is 1; it declines to 0 at *d*_max_. Field research (C. Timewell, unpublished) indicates there is an approximately exponential decay in detection probability from 1 to 0 at distances between *d*_T_ and *d*_max_. This decay can be approximated by a *topographical cover index* (*c*_T_)—a rough approximation of a site’s hilliness. Hence, at distances between *d*_T_ and *d*_max_, *p*_T_ = *e*^*c*T(*d–d*T)^. The *WildlifeDensity* user can enter an estimate of the distance at which topography starts to obscure animals.

#### Detection probability

*Initial detection probability*. At the start of the survey, the probability of detecting an animal at any distance can be modelled by combining the above models. Detectability depends on the animal’s conspicuousness, atmospheric attenuation, and vegetation and topographical cover at *d*. Hence, the detection probability at *d* is *g*(*d*), which is the product of the detection probabilities at *d* (*p*_*d*_, *p*_*b*_, *p*_v_, *p*_T_)_*d*_ minus those at *d*_max_. Therefore, *g*(*d*) reaches zero at *d*_max_ (Eq 1).


$$g(d)=a^{2}(\frac{e^{-bd}(1-c_{v}{)}^{d}∙e^{c_{T}(d-d_{T})}}{d^{2}}-\frac{e^{-bd_{max}}(1-c_{v}{)}^{d_{max}}∙e^{c_{T}(d_{max}-d_{T})}}{d_{max}^{2}})$$

Which is equivalent to:

$$g(d)={\left(p_{d}∙p_{b}∙p_{v}∙p_{T}\right)}_{d}-{\left(p_{d}∙p_{b}∙p_{v}∙p_{T}\right)}_{d_{max}}$$ (1)

Note also that as animals are detected and subsequently disregarded, progressively fewer remain available for detection in annuli nearer to the observer. This can be modelled as follows.

**Fig 5:**
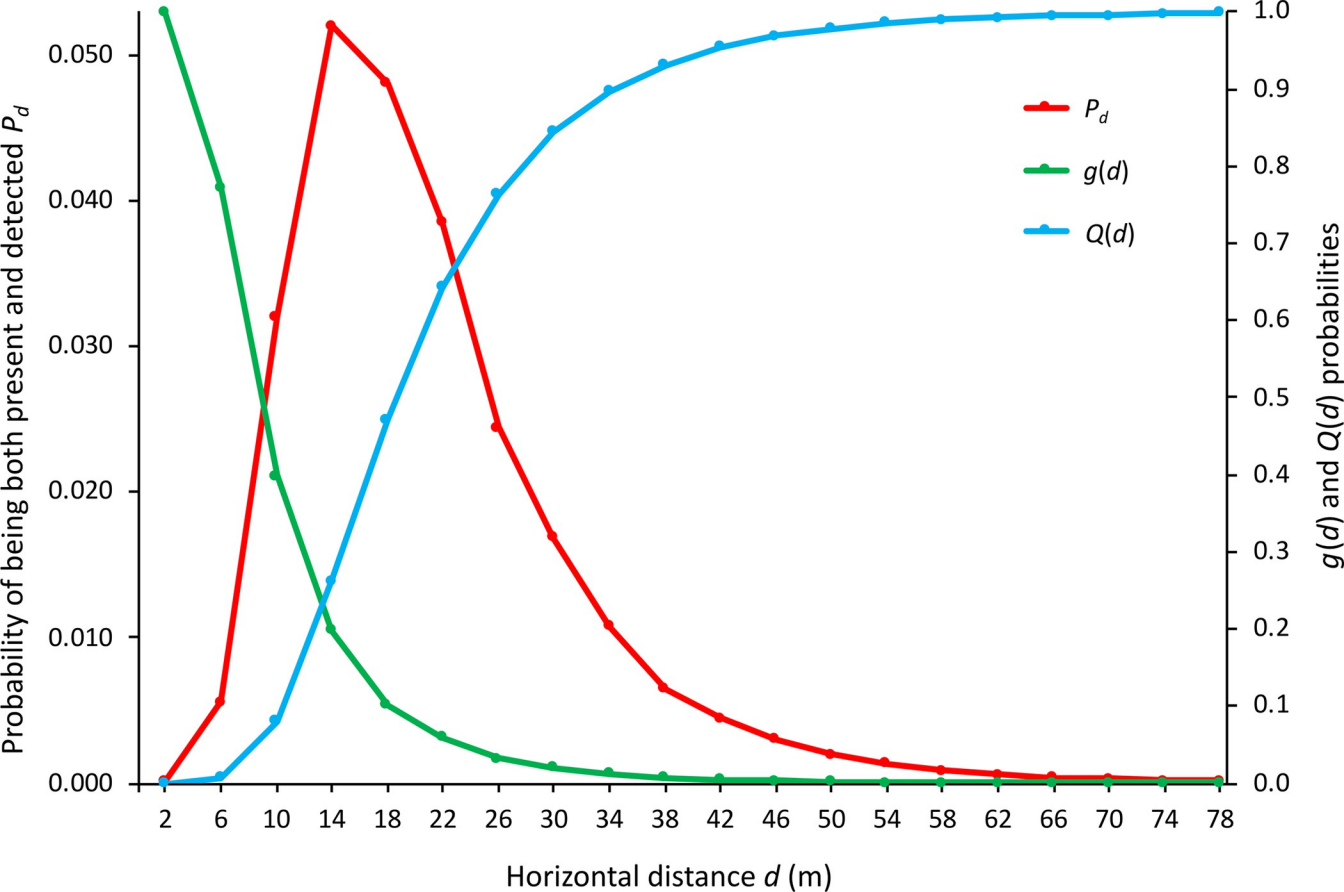
Variations in probabilities with distance. The probability of an undetected animal being present *Q*(*d*) (blue), the probability of its detection, if present *g*(*d*) (green), and the probability of being both present and detected *P*_*d*_ (red) with direct-line distance from an observer *d*, where *P*_*d*_ = *g*(*d*)*·Q*(*d + Δd*). Based on a real example of a honeyeater population in a riparian open woodland in south-eastern Australia.

*The effect of previous detections on detection probability*. All animals in the outermost observing annulus are available for detection. In all subsequent annuli, let there be a probability *Q*(*d*) that an animal is present but has remained undetected (as it was obscured or missed). As the observer advances, *Q*(*d*) decreases from 1 at *d*_max_ to 0 at *d*_min_ (Fig 5, blue line). It can be predicted by multiplying together the detection probabilities in all annuli already scanned (Eq 2):

$$Q(d)=\prod_{i=1}^{n}[1-g(d_{max}-(i-1)∙\Delta d)]$$ (2)

where *n* is the number of annuli from *d*_max_ to *d* (= (*d*_max_ *− d*)*/*Δ*d*), Δ*d* is the annulus width (set by the software), and *i* is the annulus index number (beginning at the outermost annulus).

*Overall detection probability*. The overall probability *P*_*d*_ that an as-yet undetected animal is present at *d and* is detected there will be the product of *g*(*d*) and *Q*(*d* + Δ*d*); that is, *g*(*d*) in the current annulus multiplied by *Q*(*d*) in the next annulus further out (Eq 3; Fig 5, red line):

$$P_{d}=g(d)∙Q(d+\Delta d)$$ (3)

### Estimating numbers

#### Radial data

The expected total number of detections *E*[*N*] in a radial distance class *r*_1_–*r*_2_ can now be modelled as the sum of the expected detections in all observing annuli in that class (Eq 4). In most situations, the direct-line distance *d* is equivalent to the radial distance *r*; hence, *r* is used in the following equation:

$$E[N]=DJ\;p_{a}\;n_{s}\;L\;\Delta r\sum_{i=1}^{n}\left\{r_{h}-(i-1)\Delta r\}∙g\{r-(i-1)\Delta r\}∙Q\{r-(i-2)\Delta r\right\}$$ (4)

where:

*J* = movement compensation factor (derived in the following section);

*p*_*a*_ = proportion of population observable (0 < *p*_*a*_ < 1);

*n =* number of annuli in distance class *=* (*r*_2_ *− r*_1_)*/*Δ*r*;

*n*_s_ = transect sides scanned (usually 2);

*r*_*h*_ = radial distance between observer and outer boundary of current annulus;

*r* = radial distance from observer to annulus centreline;

*g*(*r*) = mean probability of detecting an animal within the annulus of radius *r*; and

*Q*(*r*) = mean probability of an undetected animal being present in the annulus of radius *r*.

#### Perpendicular data

Because perpendicular detections are modelled in strips parallel to the transect line; several observing annuli overlap each strip except the outermost one (Fig 4). Hence, the number of detections expected in annuli between any two distances *y*_2_ and *y*_1_ is modelled by Eq 5:

$$E[N]=DJ\;p_{a}\;n_{s}\;L\;\Delta r\Delta r\sum_{j=1}^{m}∙\sum_{i=1}^{n}g\{r_{max}-(j∙n-n-i)\Delta r\}∙Q\{r_{max}+\Delta r-(j∙n-n-i)\Delta r\}$$ (5)

where:

*m* = number of distance classes in the area from *y*_max_ to *y*_min_ on each side of the transect;

*j* = position of each class in the series (outermost = 1); and

the remaining variables are as defined earlier.

Eqs 4 and 5 incorporate a movement compensation factor *J*, derived as follows.

### Compensating for animal–observer relative movement

Compared with a survey of an immobile population, detections tend to be greater when the population’s mean speed of unresponsive movement (location changes due to normal behaviour) is faster than the observer speed (e.g., many birds), as moving animals can potentially pass through the visual field more often than immobile ones.

#### Modelling population movement

Yapp [17] modelled the effects of bird movement on detections made by an observer travelling a line transect. He likened encounters between a moving observer and a mobile animal population to random intermolecular collisions, as described by the classic kinetic theory of gases for a two-dimensional situation. Greater molecular speeds increase the likelihood of collisions.

This approach was further developed by Skellam [18], who conceptualised the population as a set of molecules moving randomly in paths composed of small, straight links. The observer is like a relatively large molecule travelling in a consistent direction in the plane of the population. The ‘observer molecule’ is encircled by a *contour of interception* at the boundary of the observer’s visual field (with a radius equivalent to *r*_max_ in Fig 2). The ‘animal molecules’ (individuals and/or groups) move in random directions at a certain average velocity relative to the observer. Detections are made whenever the contour and animals make contact.

These concepts are used to develop a way of compensating for the effect of relative animal-to-observer movement on *WildlifeDensity* density estimates. Rather than using Skellam’s circular contour of interception, *WildlifeDensity* is based on modelling using D-shaped, semi-circular contours, an approach contributed by a mathematician colleague, the late Ed T. Conway. This approach provides a better cost-benefit trade-off for fieldworkers, as many more animals are usually first detected ahead than behind, while scanning a circular contour would require observers to rotate as they move ahead. Conway’s models predict the effects of population–observer relative movement on detections around the edge of the D-shaped contour. The sections below employ Conway’s derivation.

### A movement compensation model

Movement compensation requires consideration of animal and observer movement in terms of velocity (i.e., speed in a certain direction). Suppose the *observer’s velocity* has a magnitude (i.e., speed) *w* in the transect direction *θ* = 0°. Each animal has a velocity of magnitude *u* in a direction *θ* relative to the transect direction, and the animal population an average speed *ū* in random directions. Finally, each animal has a *relative velocity* in relation to the observer, of magnitude *v* and direction *θ* (for example, the relative velocity of an animal moving in the same direction and speed as the observer will be *v* = 0, *θ* = 0). Let the observer travel a line transect at a constant velocity *w* through a population of density *D* that is moving in the same plane in random directions at an average speed *ū* (in reality, animals such as flying birds may move in three dimensions). The observer scans the field ahead, which is modelled as a set of D-shaped half-annuli concentric to the detection limit of radius *r*_max_ (Fig 2). For modelling purposes, at the moment of detection each animal is assumed to be moving in a straight line. Animals will be detected within annuli of various radii. Let us focus on the number of detections expected per unit time at any radial distance *r*.

#### A D-shaped contour of interception

According to Skellam [18], at any time *t*, members of the population may differ widely in their speeds (*v*) and directions (*θ*) relative to the observer. Then, *ƒ*(*v*, *θ*, *t*) *dv·dθ* becomes the population proportion at time *t* with speeds in the interval *v ±* 0.5*dv* and directions in the interval *θ ±* 0.5*dθ*. Because the area of that part of the contour is *H*(*θ*)*v*(*t*) *dt*, the expected number of animals within that class would be *D·*ƒ(*v*, *θ*, *t*) *dv dθ·H*(*θ*)*·v*(*t*) *dt*, and the expected total number *E*{*n*} cutting the contour over a period of time from *T*_0_ to *T*_1_ will be the integral of that function over *v*, *θ* and *t* [18]:

$$E{\left\{n\right\}}_{\left(T_{0},T_{1}\right)}=D\int_{0}^{2\pi }\int_{0}^{\infty }\int_{T_{0}}^{T_{1}}f(v,\theta ,t)∙v(t)∙H(\theta )d\theta \;dv\;dt$$ (6)

**Fig 6:**
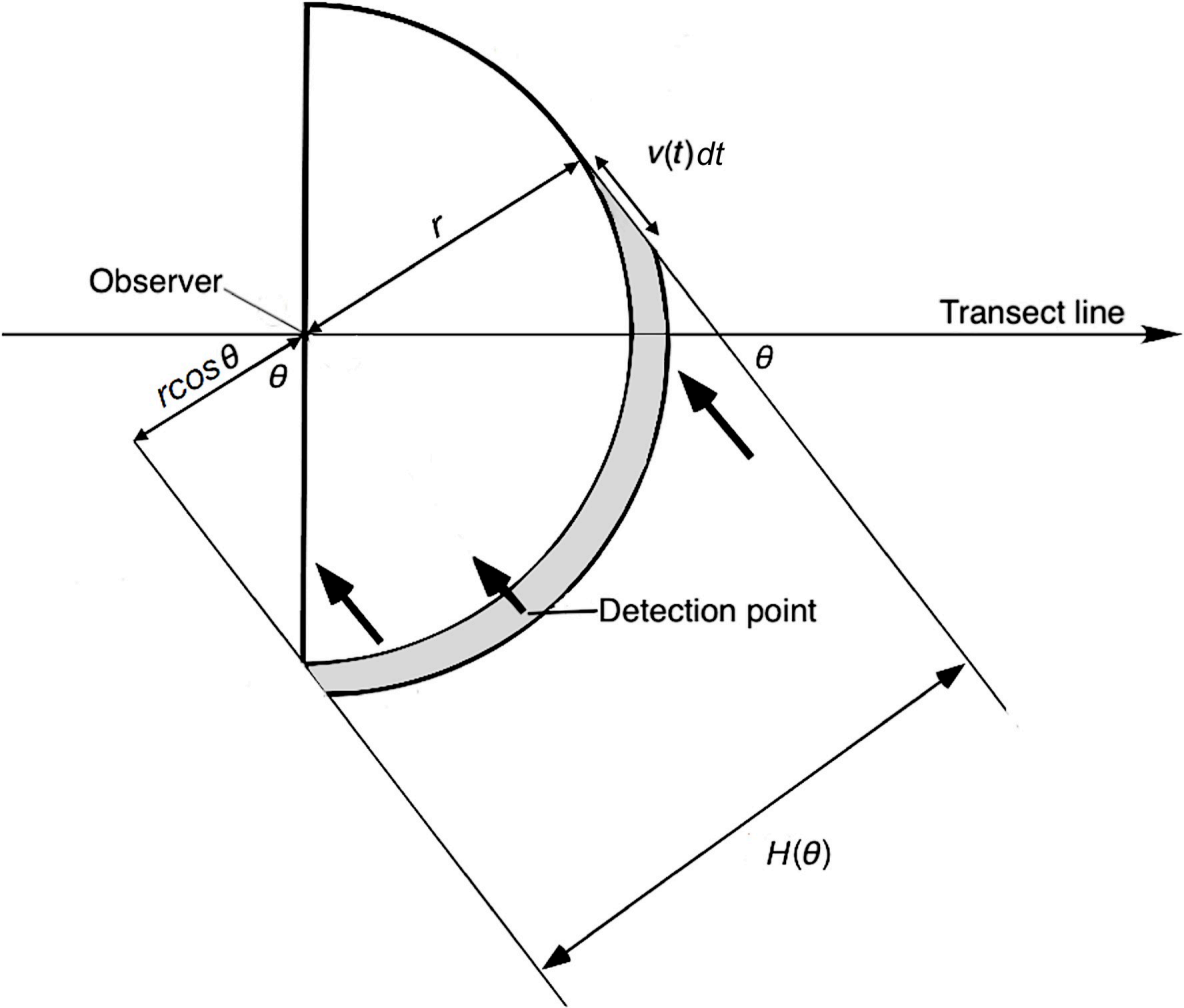
Diagram showing moving animals crossing an observer’s D-shaped contour of interception of radius *r*. Animals moving in a direction *θ* relative to the transect line are detected when they cross the contour. *H*(*θ*) is the distance between the two tangents to the contour for direction *θ*. An animal moving in this direction can only cross the shaded portion of the contour, which has a width *v*(*t*)*dt*. Any animal within this region within a small time-interval *dt* has a probability of being detected. In this example, there are three potentially detectable animals (arrows) with direction *θ*: one (middle) is detected within time *dt*, another (left) was detected a moment earlier and a third (right) is about to be detected. The speeds of each are slightly different, as represented by the arrow lengths. Such a contour will be one of many that travel with the observer along a transect line.

Using this integral, it is possible to calculate the effect of animal movement on detections. However, an integral appropriate to a D-shaped contour of interception rather than Skellam’s circular one is required (Fig 6).

#### Effects of movement on detections

If the observer has a constant velocity and the population members are classified at time *t* according to their velocities relative to the observer, so that *f*(*v*, *θ*, *t*)*dv*·*dθ* is the population proportion at time *t* with speeds within *v ±* 0.5*dv* and directions within *θ ±* 0.5*dθ*, and their distribution is unaffected by time (a reasonable assumption), then *f = f*(*v*, *θ*).

*A key relationship*. The expected total number of animals cutting the contour of interception per unit time, *E*{*n*}, for all directions and speeds, can then be given by Eq 7, which is an appropriate modification of Eq 6:

$$E\{n\}=D_{r}∙\int_{0}^{\infty }\int_{-\pi }^{\pi }f(v,\theta )∙v∙H(\theta )∙d\theta ∙dv$$ (7)

where:

*E*{*n*} = number of animals cutting the contour per unit time;

*D*_*r*_ *=* population density at *r* (detections at *r* per unit area);

*v =* animal speed relative to observer;

*θ* = animal direction relative to observer/transect;

*ƒ*(*v*, *θ*) = probability density function for *v* and *θ*; and

*H*(*θ*) *=* distance between tangents to the contour for animal direction *θ* (Fig 6).

The order of integration is unimportant, and the limits of the inner integral are more conveniently written as –*π*, *π* rather than as 0, 2*π*. All angles here are expressed in radians.

From Eq 7,

$$D_{r}=\frac{E\{n\}}{\int_{0}^{\infty }\int_{-\pi }^{\pi }f(v,\theta )v∙H(\theta )∙d\theta \;dv}$$ (8)

If the contour were a complete circle, as envisaged by Skellam, then *H*(*θ*) *=* 2*r* and this expression reduces to:

$$D_{r}=\frac{E\{n\}}{2rV}$$

Where *V* = mean velocity of animals relative to the observer and *r* = radial detection distance. However, for a D-shaped contour,

H(θ)=r(1+|cosθ|) (9)

and the integral in Eq 7 requires evaluation; i.e.,

$$I=\int_{0}^{\infty }\int_{-\pi }^{\pi }f(v,\theta )∙v∙H(\theta )∙d\theta ∙dv$$ (10)

*Terms in the integral (Eq 10)*. The value of *ƒ*(*v*, *θ*) depends on both the animals’ velocity distribution and the observer’s velocity. Animal speeds and directions are likely to be distributed independently of a stationary observer but that ceases to be the case once the observer moves forward. Any animal relatively close to and ahead of the contour of interception has some chance of being contacted. Any animal behind that region may intercept it from behind if its speed exceeds that of the observer. Whether contact occurs or not will depend on how close the animal is to the contour, how fast it and the observer are moving, in what directions it is moving, and whether the animal is detectable at that distance.

As in Skellam [18], let us make an approximation by taking the simplest distributions of the key variables. Assume that all population members have the same speed but random directions, while the observer has a constant speed and direction. Using polar coordinates, the observer’s velocity has magnitude *w* in direction *θ* = 0.

We can choose a particular angle *θ*, given the animal’s relative speed *v*, and consider the sum total of all such angles. We can then find *ƒ*(*v*, *θ*) by using the relation:

$$f(v,\theta )=f_{1}(\theta |v)∙f_{2}(v)$$ (11)

where:

*ƒ*_1_(*θ|v*) = the conditional probability density function for *θ*, given *v*. That is, the probability that an animal moving at relative speed *v* has a direction *θ*; and

*ƒ*_2_(*v*) = the probability density function for *v*; i.e., the probability of an animal having a certain relative speed.

**Fig 7:**
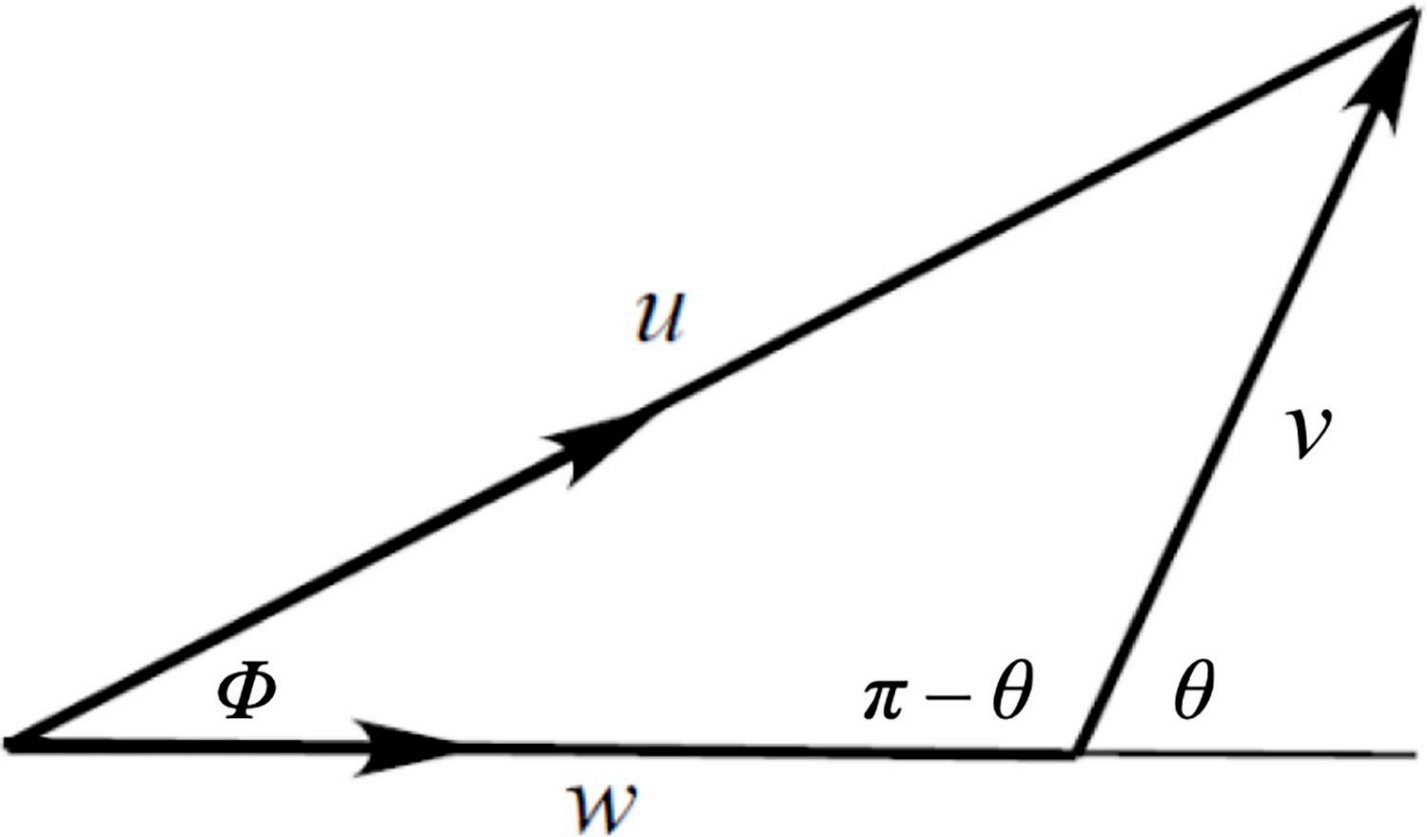
Animal–observer relative velocity. An animal moving at speed *u* and vector angle *Φ* relative to an observer moving at speed *w* along a transect has a relative velocity of speed *v* at angle *θ* (radians).


Let us begin by evaluating the probability density function *ƒ*_2_(*v*) for *v*. Fig 7 represents an observer travelling a transect at speed *w* and an animal moving at speed *u* at vector angle *Φ*. The animal’s *relative velocity v* and direction *θ* can be determined by the cosine rule (Eq 12).

$$v^{2}=u^{2}+w^{2}-2uw\;\cos\;\Phi$$ (12)

where:

*v* = animal speed relative to observer;

*u* = animal speed;

*w* = observer speed; and

*Φ* = vector angle between the animal and observer velocities.

Contacts will be made ahead of the observer with any values of *u* and *w*; animals behind are only counted if they overtake the observer, which can only happen if *u > w*. Whatever the values of *u* and *w*, the set of possible animal relative velocities *V* will have the range:

|u−w|≤V≤u+w (13)

The probability of an animal’s direction being within an angle *φ* relative to the transect line is:

Pr(|Φ|<φ)=φ/π (14)

where (0 ≤ *φ* ≤ *π* radians). Then,

$$Pr(V<v)=Pr(V^{2}<v^{2})$$

$$=Pr(u^{2}+w^{2}-2uw\;\cos\;\phi <v^{2})$$

$$=Pr(\cos\;\phi >\frac{u^{2}+w^{2}-v^{2}}{2uw})$$

$$=Pr(|\phi |<\mathrm{arcos}\frac{u^{2}+w^{2}-v^{2}}{2uw})$$

$$=\frac{\mathrm{arcos}\frac{u^{2}+w^{2}-v^{2}}{2uw}}{\pi }$$ (15)

with the numerator thus replacing *φ* in Eq 14.

The probability density function for *v* is *ƒ*_2_(*v*); i.e., the population proportion with relative velocities between *v* − 0.5*dv* and *v +* 0.5*dv*, and is obtained by differentiating with respect to *v*. Thus,

$$f^{2}(v)=\frac{d}{dv}\left\{\frac{\mathrm{arcos}\frac{u^{2}+w^{2}-v^{2}}{2uw}}{\pi }\right\}$$

Hence,

$$f_{2}(v)=\frac{2v}{\pi \sqrt{4u^{2}w^{2}}-{\left(u^{2}+w^{2}-v^{2}\right)}^{2}}$$

$$=\frac{2v}{\pi \sqrt{\left\{v^{2}-{\left(u-w\right)}^{2}\}\{{\left(u+w\right)}^{2}-v^{2}\right\}}}$$ (16)

for |u−w|≤v≤u+w, and *ƒ*_2_(*v*) *=* 0 for all other values of *v*.

We now need an expression for *ƒ*_1_(*θ|v*), the probability density function for *θ*, given *v*. Notice that the angle *θ* in Fig 6 will always satisfy *π/*2 *< θ < π*. From Fig 7, because all animals moving at relative speed *v* have, in effect, a single direction *θ* relative to the observer, then according to the cosine rule (Eq 12),

$$\cos\;\theta =\frac{u^{2}-w^{2}-V^{2}}{2wV}$$ (17)

and

$$|\theta |=\mathrm{arcos}\frac{u^{2}-w^{2}-V^{2}}{2wV}$$ (18)

This tells us that *ƒ*_1_(*θ|v*) is not a continuous function; once *v* is given as *V*, *|θ|* is uniquely determined. This can be expressed in terms of a delta function for *ƒ*_1_(*θ|v*):

$$f(\left|\theta ||v\right)=\delta \left(|\theta |-\mathrm{arcos}\frac{u^{2}-w^{2}-v^{2}}{2wv}\right)$$ (19)

So,

$$f_{1}(\theta |v)=0.5\left\{\delta (\theta -\mathrm{arcos}\frac{u^{2}-w^{2}-v^{2}}{2wv})+\delta (\theta +\mathrm{arcos}\frac{u^{2}-w^{2}-v^{2}}{2wv})\right\}$$ (20)

The width of the contour of interception is *H*(*θ*). From the mean value theorem (see [18], p. 392), the effect of using a D-shaped contour is that H(θ)=r(1+|cosθ|), as per Eq 9, with a value between *r* and 2*r* (see Fig 6), where *r* is the radius of the contour of interception.

#### Function evaluation

We can now go ahead and evaluate the integral in Eq 7. Then, using Eq 11, the integral becomes:

$$I=D∙\int_{0}^{\infty }\;\int_{-\pi }^{\pi }f_{2}(v){∙f}_{1}(\theta |v)∙v∙H(\theta )d\theta \;dv$$ (21)

*The inner integral*. The inner integral, by the property of *δ*-functions, is now (from Eq 20):

$$\int_{-\pi }^{\pi }f_{2}(v){∙f}_{1}(\theta |v)∙v∙H(\theta )d\theta =0.5f_{2}(v)∙v∙H(\mathrm{arcos}\frac{u^{2}-w^{2}-v^{2}}{2wv})+0.5f_{2}(v)∙v∙H(-\mathrm{arcos}\frac{u^{2}-w^{2}-v^{2}}{2wv})$$ (22)

Then, using the D-shaped contour (Eq 9), this inner integral is simply:

$$f_{2}(v)\cdot v\cdot r\cdot \left(1+\left|\frac{u^{2}-w^{2}-v^{2}}{2wv}\right|\right)$$ (23)

*The whole integral*. Consider now what happens as an observer moves forward along a transect at speed *w*, searching around a D-shaped contour while travelling through a population moving at a mean speed *ū* in a variety of directions. As we have modelled the situation, potentially detectable animals can be classified according to their relative speeds: 1) those with speeds greater than the observer (*u > w*) may contact the contour of interception from any direction, including by overtaking from behind, 2) animals with speeds less than that of the observer (*w > u*) are detected as the observer passes by and 3) those with similar speeds (*u = w*) have a lower probability of detection, as the paths of the animal and contour are less likely to cross than in the other cases. Although real-world animal and observer speeds can vary in a highly complex way, this pattern is sufficiently representative. The integration process from here on depends on the relative magnitudes of *u* and *w*.

*Case 1*: *When u > w*, then |*u − w| ≤ V ≤ u + w*. Because *ƒ*_2_(*v*) is zero outside the range *|u − w|* → *u + w*, using Eq 23, the whole integral becomes:

$$I=\int_{\left|u-w\right|}^{u+w}f_{2}(v)∙v∙r\left(1+\left|\frac{u^{2}-w^{2}-v^{2}}{2wv}\right|\right)$$

$$=\int_{\left|u-w\right|}^{u+w}\frac{2v}{\pi \sqrt{\left\{v^{2}-{\left(u-w\right)}^{2}\}\{{\left(u+w\right)}^{2}-v^{2}\right\}}}∙v∙r\left(1+\left|\frac{u^{2}-w^{2}-v^{2}}{2wv}\right|\right)dv$$

$$=\frac{2r}{\pi }\int_{\left|u-w\right|}^{u+w}\frac{v^{2}(1+|\frac{u^{2}-w^{2}-v^{2}}{2wv}|)}{\sqrt{\left\{v^{2}-{\left(u-w\right)}^{2}\}\{{\left(u+w\right)}^{2}-v^{2}\right\}}}dv$$ (24)

*Case 2*: *When w > u*, then *w* − *u* ≤ *V* ≤ *u* + *w*. The lower limit of integration in Eq 24 now becomes *w–u*; thus:

$$I=\frac{2r}{\pi }\int_{w-u}^{u+w}\frac{v^{2}(1+\left|\frac{u^{2}-w^{2}-v^{2}}{2wv}\right|)}{\sqrt{\left\{v^{2}-{\left(u-w\right)}^{2}\}\{{\left(u+w\right)}^{2}-v^{2}\right\}}}dv$$ (25)

*Case 3*: *When u = w*, then Eq 25 becomes:

$$I=\frac{2r}{\pi }\int_{0}^{2u}\frac{v^{2}(1+\left|\frac{-v^{2}}{2uv}\right|)}{\sqrt{v^{2}\{{\left(2u\right)}^{2}-v^{2}\}}}dv$$

$$=\frac{2r}{\pi }\int_{0}^{2u}\frac{v^{2}(1+\left|\frac{v}{2u}\right|)}{\sqrt{v^{2}\{{\left(2u\right)}^{2}-v^{2}\}}}dv$$ (26)

Only in Case 1 (Eq 24, fast animals where *u* > *w*) is animal movement highly likely to bring significantly more individuals into the path of an observer, thereby requiring movement compensation. Once values for *u*, *w* and *r* are chosen, the improper integral there can be evaluated numerically. *D*_*r*_ from Eq 7 can also be calculated as *E*{*n*}/*I*.

#### A movement compensation factor

To compensate for the effect of animal movement on detections along a line transect, we need to compare the number actually detected with the number expected for an immobile population.

*Expected contacts per unit time*. Let *k* be the animal-to-observer relative speed, where *k = u*/*w*. Then, using Eq 24, we have:

$$I=\frac{2r}{\pi }\int_{w(k-1)}^{w(k+1)}\frac{v^{2}(1+\left|\frac{(k^{2}-1)w^{2}-v^{2}}{2wv}\right|)}{\sqrt{\left\{{v^{2}-w^{2}(k-1)}^{2}\}\{w^{2}{\left(k+1\right)}^{2}-v^{2}\right\}}}dv$$ (27)

If we then let *v = ws*, where *s* is the ratio between *w* and *v*, then:

$$I=\frac{2r}{\pi }\int_{\left|k-1\right|}^{k+1}\frac{w^{2}s^{2}(1+\left|\frac{k^{2}-1-s^{2}}{2s}\right|)}{\sqrt{\{{s^{2}-(k-1)}^{2}\}\{{\left(k+1\right)}^{2}-s^{2}\}w^{4}}}wds$$

$$=\frac{2rw}{\pi }\int_{\left|k-1\right|}^{k+1}\frac{s^{2}(1+\left|\frac{k^{2}-1-s^{2}}{2s}\right|)}{\sqrt{\left\{{s^{2}-(k-1)}^{2}\}\{{\left(k+1\right)}^{2}-s^{2}\right\}}}ds$$ (28)

For a given mean animal speed *ū* and observer speed *w*, let *J* be the ratio between the number of detections and that expected for immobile animals (*ū =* 0); i.e.,

$$J=\frac{detections\;expected\;at\;animal\;speed\;\bar{u}}{detections\;expected\;at\;animal\;speed\;0}$$ (29)

*Contacts if animals immobile*. If all animals were immobile, the number of contacts *E*{*n*} expected on the perimeter of a D-shaped contour of radius *r* per unit time is expected to equal the density at *r* multiplied by the area swept out per unit time; i.e.,

$$E\{n\}=D_{r}∙2rw$$ (30)

#### The movement compensation factor *J*

Replacing the numerator in Eq 29 with Eq 28, and the denominator with Eq 30, we get:

$$J=D_{r}\frac{2rw}{\pi }\int_{\left|k-1\right|}^{k+1}\frac{s^{2}(1+\left|\frac{k^{2}-1-s^{2}}{2s}\right|)}{\sqrt{\left\{{s^{2}-(k-1)}^{2}\}\{{\left(k+1\right)}^{2}-s^{2}\right\}}}ds/D_{r}∙2rw$$ (31)

Then, for any given value of *k*, we get Eq 32:

$$J=\frac{1}{\pi }\int_{\left|k-1\right|}^{k+1}\frac{s^{2}(1+\left|\frac{k^{2}-1-s^{2}}{2s}\right|)}{\sqrt{\left\{\left[s^{2}-{\left(k-1\right)}^{2}][{\left(k+1\right)}^{2}-s^{2}\right]\right\}}}ds$$ (32)

This improper integral can now be evaluated numerically. It is improper at both ends, but convergent. Notice that the value of *J* is independent of both the population density *D* and the radial detection distance *r—*its magnitude depends only on the population speed *ū* and observer speed *w*.

Then, at *any* radial distance *r*,

$$D_{r}=\frac{E\{n\}}{2rwJ}$$ (33)

If an observer travels a transect of length *L* in time *T*, and detects individuals at a certain frequency *ƒ*_*r*_ at various radial distances *r*, then the number of detections per unit time at *r*, *n*_*r*_, is given by:

$$n_{r}=\frac{f_{r}}{T}$$ (34)

In the density estimator represented by Eq 33, *J* serves as a *movement compensation factor*. Because *J* is a function of the population–observer relative speed and is independent of *r*, it can be applied to reduce the observed number of detections of a mobile population to that expected for an immobile one. *J* itself is dimensionless.

*Calculating animal speed ū*. For modelling purposes, the animal and observer speeds are approximated as constants. Given that observers endeavour to move at a constant speed, *w* can be estimated as the transect length divided by the travel time (*w* = *L*/*T*, m/min). Then, *ū* is calculated by classifying the main *behavioural types i* of the species (e.g., resting, flying, walking), quantifying the *proportions of time spent in each behaviour p*_*i*_, and estimating the *average horizontal speed of each behaviour u*_*i*_ (Eq 35).


$$\bar{u}=\sum \left(p_{i}∙u_{i}\right)$$ (35)

Estimation of *p*_*i*_ and *u*_*i*_ requires independent study of the target species under the survey conditions (same time and environment). For example, an observer may spend several hours recording behaviours at 10 s intervals (e.g., *i =* stationary, walking, travelling, fleeing) to establish values for *p*_*i*_. Then, the average speeds of these behaviours are estimated to establish values for *u*_*i*_. For example, a bird’s flights can be timed and the distances measured to obtain *u*_travelling_. Some movement speeds (e.g., flight) of particular species may be available in existing research. Sometimes, an approximation based on a known species may suffice. Further guidance is provided in the *WildlifeDensity* manual [15]. We have not yet incorporated a measure of variance in *ū*. This may be a topic of further research.

**Fig 8:**
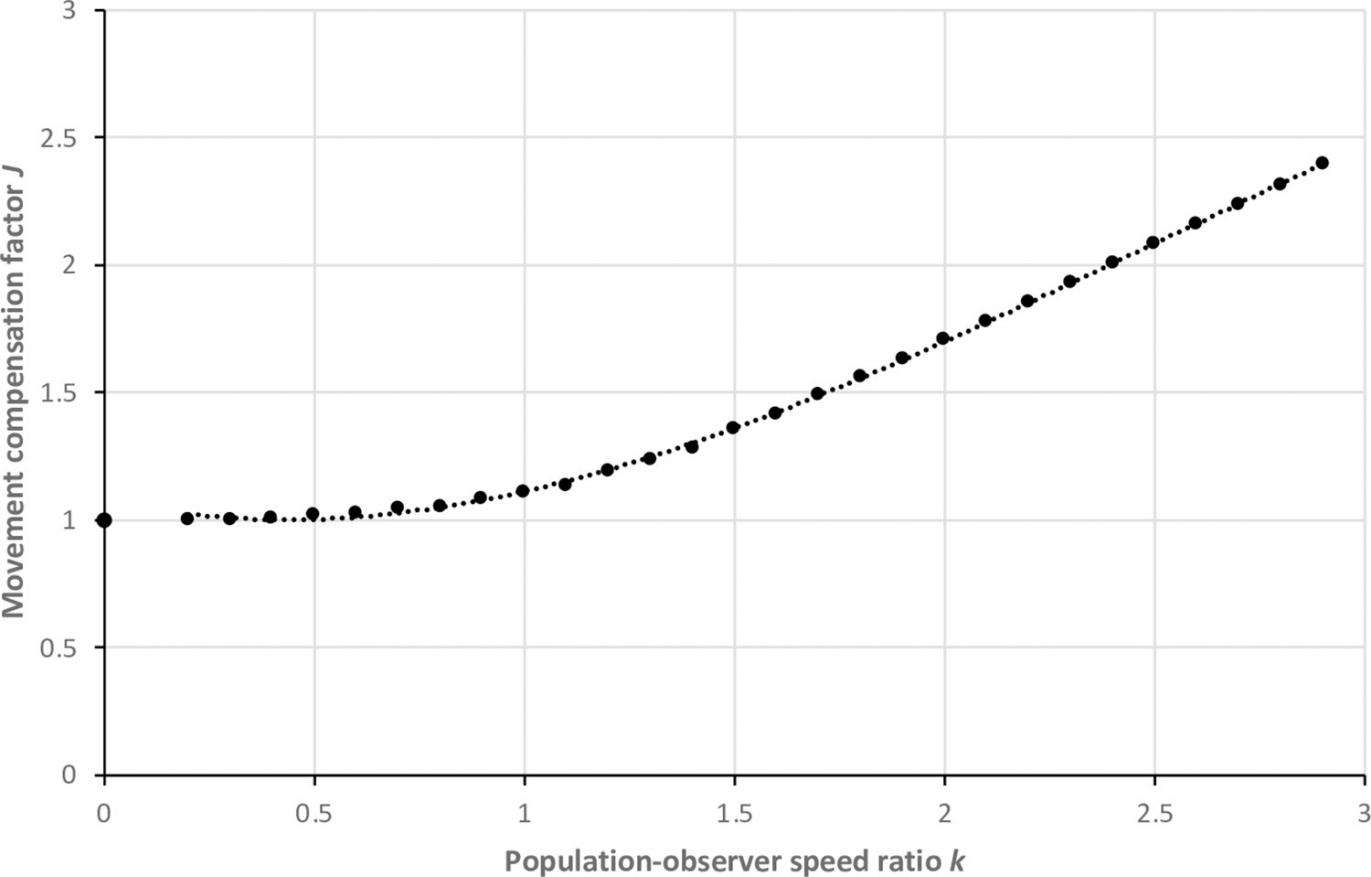
Relationship between the movement compensation factor *J* and population-observer speed ratio *k*. Dots represent the numerical integration of Eq 32, while the dotted line represents the approximation provided by Eq 36. At *k* < 0.5, the effect of *k* on detections is negligible; while at higher ratios, expected detections are much greater (e.g., 2.5 times at *k* = 3), suggesting that movement compensation is essential in some surveys.

*Evaluating J*. Numerical integration of Eq 32 produces *J* values for values of *k* (Fig 8). Provided that an estimate of *ū* is supplied, *WildlifeDensity* calculates *J* and includes it with the results of each density estimate. If an investigator requires a *J* value independently of *WildlifeDensity*, they can first calculate *k* (= *ū*/*w*). At *k* = 0–0.3, *J* is effectively 1. For *k* = 0.3–3.0, *J* can be closely approximated using a simple polynomial:

$$J=-0.068k^{3}+0.474k^{2}-0.324k+1.069$$ (36)

For *k* ≥ 3, the relationship approaches linearity, where:

J=0.82k (37)

### Density estimation

#### Setting up an analysis

In the *WildlifeDensity* program, the *Method* and *Sample Details* menus are used to enter details such as survey type, transect length and time taken, and population speed *ū*. The detection data (distances and group sizes) are entered in the *Observations* menu manually or by copying from a text file. In the *Options* menu, one can set up an analysis by entering a class interval width and number of iterations to be used in bootstrapping (say, 500–1000). The initial values of parameters *D*, *c*, *a* and *d*_max_ and their step sizes are chosen automatically, or can be specified if they are already known. Calculations are then initiated from the *Estimate* menu. Further advice is available in the guide provided with the software.

#### Computational summary

*WildlifeDensity* was originally written in Fortran and revised using a combination of C, C++ and Objective C to run on MacOS systems. Processing begins by incorporating survey data such as the number of transect sides searched (1 or 2) and the transect lengths and times taken. The maximum detection distance is estimated from the data or can be set manually. The program uses the relevant objective function model (Eq 7 or 8) to determine the densities expected in distance classes across the range, with classes usually subdivided into multiple observing annuli. When sample sizes are large (> 250), estimates are made of three parameters: *D* (density), *a* (conspicuousness) and *c* (lateral cover); the latter two ‘shape parameters’ affect the rate of change in detectability with distance and, hence, the form of the detection vs distance frequency distribution. With smaller samples, the mean and standard error of *D* and one shape parameter are estimated, together with an approximation to the other shape parameter.

Within each distance class, the numbers of detections expected in each arc are totalled and compared with the numbers observed in that class. A total sum of squares of the differences between the estimated and observed detection numbers in the various classes is then calculated. This is followed by a numerical function minimization process (Section *Function minimisation*) for each of the main parameters in turn to try to minimise the differences between the observed and estimated detections.

To start the minimisation process, pre-set initial parameter estimates are normally used, based on previous *WildlifeDensity* analyses of representative species. Alternatively, suitable initial values can be entered. A function minimization process using the original data then provides the first set of parameter estimates. The result is supplemented by multiple resampling of the original data with replacement, or bootstrapping, for a pre-determined number of iterations (e.g., 500). In each case, a stepwise procedure varies the initial values of the expediently modified parameter *D·*2*L* and the two main shape parameters. The program then progressively minimizes the differences and retains the resulting parameter estimates. This produces several sets of parameter estimates with multiple cases in each, every set of which is usually approximately normally distributed (as assessed by Shapiro-Wilk tests). These are used to calculate the final values of parameters *D*, *a* and *c* and their standard errors.

#### Function minimization

For development purposes, the *WildlifeDensity* program was built from several subroutines that execute the iteration calculations for each dataset. For instance, to drive the bootstrapping, a set of pseudo-random number generator subroutines is first seeded from the original field data, then used to select the subsets of the bootstrapping. Case selection is then at random with replacement, based on the relevant pseudo-randomly selected index number, which may be any number from 1 to the total number of detections in the relevant dataset.

Parameter estimation follows a modification of the classic Nelder-Mead ‘downhill’ simplex method of function optimization [19, 20]. This uses algorithms based on MINIM software [21], which were developed by staff of the Commonwealth Scientific Industrial Research Organisation (Sydney, Australia) and the Rothamsted Experimental Station (UK). It is a direct-search method that estimates the main parameters in turn, beginning with *D*. The data are analysed in distance classes in order from the furthest class to the closest one (usually ending at 0 m). The process begins by setting up a simplex: a conceptually conceived geometrical shape consisting of *n* + 1 test points, or vertices, in *n* dimensions (where *n* is the number of parameters being estimated), initially spaced well apart and linked by straight lines. The coordinates of the initial points are derived from the data and the initial step sizes. The program then progressively seeks to reduce the differences between observed and estimated detection numbers through a progressive function minimization process, modifying the simplex as it goes. All variables and numerical arrays are calculated at double precision.

The main routine varies the values of *D*, *a* and *c*, using the Nelder-Mead approach to minimise the overall difference between the modelled and observed detection data. An algorithm in *n* + 1 dimensions maintains a set of test points spatially arranged as a simplex of *n* + 1 points in space. At each test point, the original point is replaced with a new one, the simplest approach being to replace the ‘worst’ point with a new point reflected mathematically through the centroid (i.e., geometric centre) of the remaining *n* points of the simplex. If the new point is better than the best current point, then an exponential expansion along the line between them is followed. The reflection side of the centroid, then the other side, may also be contracted in length. Or, if the new point is not much better than the previous value, then the overall simplex is shrunk towards a better one.

The modified simplex is tested for its degree of agreement with previous results, and a new set of parameter values is derived. The process is repeated until comparison with a predetermined stopping criterion shows that the function minimization process has reached a stage where the function difference between modelled and observed detections has become very small and acceptably close. The estimate of *D* is retained, together with final estimates of the shape parameters *a* and *c*. If an acceptably close result is not produced after 750 iterations, the search for a minimum is terminated and the analysis treated as unsuccessful. It may then be appropriate to increase the sample size by collecting and processing additional field data.

If, as has been our usual experience, the analysis proceeds until an acceptable minimum is reached (convergence), a set of bootstrapped data is selected and treated as above to produce another set of parameter values. This entire process is repeated until the predetermined number of bootstraps is complete. Ultimately, the accumulated estimates are used to calculate final estimates of the means and standard errors of the main parameters. These are output, along with an overall detectability coefficient *S* that delivers a single measure of detectability, the observed and calculated frequency distributions, and an approximation to the detection probability at the transect line *g*(0).

### Using stratification with line transect surveys

Line transect surveys can sometimes be made more precise by dividing the environment or population into distinctive groups, or *strata*, that differ in some attribute likely to affect detectability, commonly the vegetation type (*habitat*). In each stratum, the transect length and detection data are recorded separately. Data from different strata are then analysed separately (e.g., by *WildlifeDensity*). The resulting density estimates and variances are then combined after weighting each according to the proportion of transect spent in that stratum type.

Thus, if *L* strata are being combined, the weighting factor for the *h*^th^ stratum will be *N*_*h*_/*N*, where *N*_*h*_ is the transect length spent in the *h*^th^ stratum and *N* is the total transect length. The density estimate of the *h*^th^ stratum *is* ${\bar{y}}_{h}$. The overall mean density (${\bar{y}}_{st}$) of the combined strata is then given by Eq (38):

$${\bar{y}}_{st}=\sum_{h}^{L}\frac{N_{h}}{N}{\bar{y}}_{h}$$ (38)

The derivation of a variance $var({\bar{y}}_{st})$ of the mean of a stratified sample cannot simply rely on proportional allocation because their sample sizes (detection numbers) *n*_1_, *n*_2_,*…n*_*L*_ are random variables, the values of which were obtained *en route* during sampling. Instead, *poststratification methods* are appropriate when determining variances [22]. If $\sigma_{h}^{2}$ is the variance of the population in the *h*^th^ stratum, the estimated overall variance of the mean of the combined stratified sample will be approximately that given by Eq (39), with the standard error being its square root.


$$\hat{var}{(\bar{y}}_{st})\approx \frac{N-n}{nN}\sum_{h=1}^{L}(\frac{N_{h}}{N})\sigma_{h}^{2}+\frac{1}{n^{2}}\left(\frac{N-n}{N-1}\right)\sum_{h=1}^{L}\frac{N-N_{h}}{N}\sigma_{h}^{2}$$ (39)

## Evaluation

While the mathematical derivation is somewhat complex, the survey protocol and software usage procedures are relatively straightforward. Ultimately what matters is that the technique can be demonstrated to work in practice. This section examines *WildlifeDensity*’s capacity to 1) estimate the densities of ‘benchmark’ terrestrial populations for which density is already known, 2) deal with inflated detection numbers in line transect surveys of highly mobile populations, and 3) estimate density in a fairly difficult scenario—that of a small, highly mobile songbird occurring at low density in a forested site with heavy cover. Comparisons are also made with conventional *Distance* analysis.

### Test 1: Known-density populations

**Method.** *WildlifeDensity* was applied to existing line-transect distance sampling datasets for which the population density and movement rate were known [8, 23, 24, J. Wischusen, unpubl. data]. Table 1 summarises these datasets, with the methods used to determine the true densities shown in the right column. Three surveys were of inanimate, immobile objects (including one by helicopter) and two were of relatively immobile herbivores (*ū* = 5 m/min). *WildlifeDensity* estimates were made using both radial and perpendicular detection distances and compared with those of conventional *Distance* analysis.

**Table 1:** Known-density datasets used to evaluate *WildlifeDensity*. Survey 2 was aerial; the others were walked. Surveys 3 and 5 were each undertaken five times.

No.	Population	Location [source]	Habitat	Transect length (km)	Animal speed *ū* (m/min)	Numbers detected	Known density source; notes
Immobile populations							
**1**	Wooden stakes	Logan, UT [23]	Sagebrush meadow	1.0	0	68	Objects placed by design
**2**	Feral pig carcasses	Arnhem Land, NT [24]	Open floodplain	348.0	0	51	Known number culled from aircraft
**3a–e**	Eyelevel tree tags	Clonbinane, VIC [J. Wischusen, unpubl. data]	Open forest	2.0	0	177, 212, 252, 260, 199	Objects placed by design; identical scenario, 5 observers
Relatively immobile populations							
**4**	Red kangaroo	Kinchega, NSW [8; unpubl. data]	Fenced low shrubland	89.3	~5	200	Preliminary drive count in open, elongated site
**5a–e**	Red kangaroo	Tidbinbilla, ACT [8; unpubl. data]	Open forest enclosures	21.8	~5	242, 197, 166, 204, 101	Tagged, habituated population in reserve; 5 surveys at different times (5c, e by spotlight at night)

### Results

Table 2 shows the density estimates obtained using the three methods. Shown also are the standard error (SE), bias, and root mean squared error (RMSE)—a combined measure of SE and bias expressed as a percentage. For all variables except density, lower values indicate superior estimation performance.

**Table 2:** Density estimates (est. D), standard errors (SE), bias and root mean squared errors (RMSEs) obtained by three methods of analysis of line-transect surveys of known-density populations. *Radial data were not available for Surveys 1 and 2. ^†^Densities for surveys 2 and 4 are per km^2^.

Survey	True D	*WildlifeDensity*–radial				*WildlifeDensity*–perpendicular				*Distance*–perpendicular			
	(no./ha)	Est. D	SE	Bias (%)	RMSE (%)	Est. D	SE	Bias (%)	RMSE (%)	Est. D	SE	Bias (%)	RMSE (%)
	Immobile populations												
**1**	37.5	.*	.	.	.	34.5	3.40	-7.9	12.0	36.1	4.32	-3.7	12.1
**2**	2.8^†^	.*	.	.	.	3.0	0.48	6.8	18.4	2.5	0.17	-12.1	13.6
**3a**	14.6	14.7	0.83	0.3	5.7	14.1	1.21	-3.7	9.1	14.1	0.67	-3.5	5.7
**3b**	14.6	14.4	0.66	-1.1	4.7	14.0	0.93	-4.2	7.6	15.1	0.75	3.2	6.0
**3c**	14.6	16.6	1.31	14.0	16.0	14.7	0.69	1.0	4.8	14.8	0.40	1.3	3.0
**3d**	14.6	16.0	0.71	9.8	10.9	15.9	0.76	9.2	10.6	15.9	0.22	9.2	9.3
**3e**	14.6	15.2	0.79	3.9	6.7	14.0	1.01	-4.1	8.1	14.4	0.55	-1.6	4.1
**Mean 3a-e**	**14.6**	**15.4**		**5.4**	**8.8**	**14.5**		**-0.4**	**8.0**	**14.9**		**1.7**	**5.6**
**Mean 1–3**	**16.2**					**15.8**		**-0.4**	**10.1**	**16.1**		**-1.0**	**7.7**
Relatively immobile populations													
**4**	14.0^†^	13.7	0.99	-2.3	7.5	13.6	1.24	-2.6	9.2	13.8	1.25	-1.4	9.0
**5a**	2.2	2.2	0.20	-2.7	9.4	2.1	0.19	-7.7	11.5	2.0	0.14	-10.8	12.5
**5b**	2.7	2.3	0.25	-14.4	17.1	2.0	0.32	-26.9	29.4	1.6	0.13	-39.5	39.8
**5c**	2.7	2.5	0.20	-8.5	11.3	2.0	0.23	-25.8	27.2	2.0	0.13	-28.0	28.5
**5d**	2.0	2.4	0.19	23.2	25.1	1.9	0.18	-6.1	10.1	1.8	0.15	-8.1	11.1
**5e**	2.0	2.0	0.22	-0.5	11.1	1.7	0.19	-15.2	17.9	2.0	0.35	-0.5	17.7
**Mean 4–5**	**4.3**	**4.2**		**-0.9**	**13.6**	**3.9**		**-14.0**	**17.6**	**3.9**		**-14.7**	**19.8**
**Mean 3–5**	**9.0**	**9.3**		**2.0**	**11.4**	**8.7**		**-7.8**	**13.2**	**8.9**		**-7.2**	**13.3**

Shapiro-Wilk tests indicated that the frequency distributions of densities estimated by the three methods were not significantly different from normal. Paired-samples *t*-tests were then used to compare estimated and known densities in the 11 surveys for which all three methods could be applied (Surveys 3a–5e). There were no significant differences between the known densities and those estimated by *WildlifeDensity* using radial (*n* = 11, *t* = –1.350, *p* = 0.207) or perpendicular data (*n* = 11, *t* = 1.361, *p* = 0.203) or by *Distance* using perpendicular data (*n* = 11, *t* = 0.553, *p* = 0.593).

#### Bias

In Surveys 1 and 2—immobile populations for which radial data were unavailable—perpendicular *WildlifeDensity* data produced density estimates with lower bias (mean = –0.6%) than *Distance* (–7.9%). With the immobile population data, all three techniques can only be compared using Survey 3. Here, *WildlifeDensity* perpendicular and *Distance* perpendicular performed similarly (–0.4% and 1.7%, respectively) and slightly better than *WildlifeDensity* radial (5.4%). For the relatively immobile populations (Surveys 4 and 5), *WildlifeDensity* radial outperformed *WildlifeDensity* perpendicular and *Distance* perpendicular (–0.9%, –14.1% and –14.7%, respectively). Comparing all three methods across all datasets (Surveys 3–5), a similar result was obtained, with *WildlifeDensity* radial outperforming *WildlifeDensity* perpendicular and *Distance* perpendicular (2.0%, –7.8% and –7.2%, respectively). These results suggest that all three techniques show acceptable levels of accuracy with data from largely immobile populations.

#### Error

While *WildlifeDensity* estimates generally had less bias than *Distance* ones, most (but not all) of their SE values were higher, suggesting that *WildlifeDensity* may often be more accurate but less precise. We consider the RMSE as our most useful single indicator of estimation quality as it combines the SE and bias, indicating both precision and accuracy. With the completely immobile populations (Surveys 1–3), *WildlifeDensity* perpendicular performed similarly to *Distance* (mean RMSEs = 10.1% and 7.7%, respectively). When comparing all three techniques on an immobile population (possible with Surveys 3a-e only), similar mean RMSEs were obtained (8.8%, 8.0%, 5.6%, respectively). With the relatively immobile kangaroo populations (Surveys 4–5), *WildlifeDensity* radial performed slightly better than *WildlifeDensity* and *Distance* perpendicular (13.6%, 17.6%, 19.8%, respectively). Overall, the three methods showed comparable, acceptable error levels with populations of low or no mobility.

As there are no suitable datasets on highly mobile populations of known density, we undertook two additional tests. Test 2 investigates the movement compensation ability of *WildlifeDensity*, while Test 3 estimates density in a breeding population of a low-density, inconspicuous and highly mobile forest songbird.

### Test 2: Movement compensation

#### Method

Test 2 examines whether the movement compensation factor (*J*) can adequately allow for the effect of high animal–observer relative movement. As it is impossible to vary animal speeds in a suitable experimental fashion, we instead varied the observer speed. Two separate surveys were conducted, where observers walked transects multiple times at a variety of speeds while searching for highly mobile Australian songbirds: 1) the grey fantail (*Rhipidura albiscapa*) and 2) red wattlebird (*Anthochaera carunculata*). Both move about forest habitats continually as they forage.

Estimates of *ū* were obtained by measuring the speed of each behaviour type and the proportion of time spent in each. Speed estimation required techniques such as measuring the times and distances of flights between trees. Eq 35 was used to estimate overall speeds of *ū* = 116 m/min for fantails and *ū* = 66 m/min for wattlebirds.

Single observers (Survey 1 = R. Plant, Survey 2 = J. Gibbens) conducted line transect counts of birds (fantail, wattlebird) while walking forest paths (lengths = 1000 m, 1370 m) at a variety of approximately constant speeds (ranges *=* 15–100 m/min, 17–80 m/min), making 372 and 898 detections, respectively. Observer–animal distances were not required for this task. Eqs 36 and 37 were used to calculate *J-*values for each observer speed class, which were used to adjust the detection numbers (= detections/*J*). We hypothesised that a faster-moving observer would make fewer detections because birds would have less time to leave and re-enter their visual field and be detected again. It is also possible that a faster-moving observer makes fewer detections because they have less time to scan the visual field and/or because their increased conspicuousness flushes animals. To minimise this, we restricted the range of speeds to those where the observer was capable of adhering to the field protocol. We believe flushing to have been minimal as the birds at the study site stayed well above ground level and appeared unresponsive to people below.

#### Results

**Fig 9:**
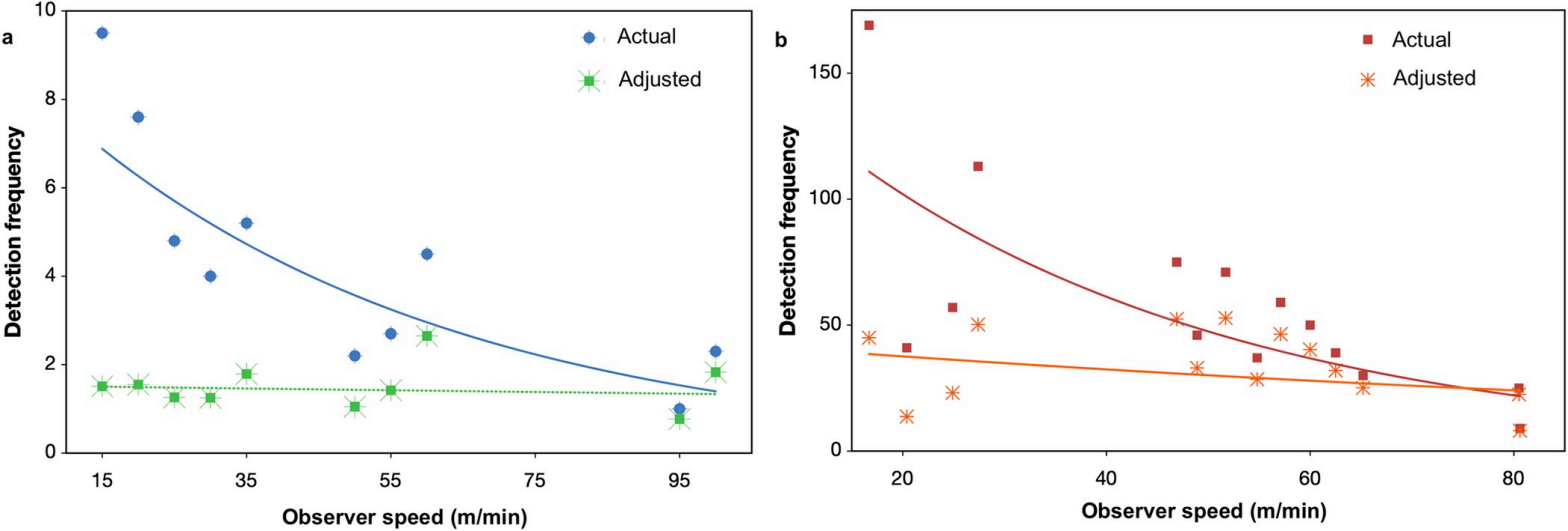
Relationships between observer speed and detection numbers during transect surveys of two highly mobile birds: the grey fantail (a) and red wattlebird (b). Star-like symbols represent adjusted numbers (after division by *J*). The trendlines for unadjusted data are logarithmic and steeply decreasing, while those for the adjusted data are approximately linear and flat (with gradients not significantly different to zero), showing that adjustment by *J* can compensate for the effect of relative movement.

Fig 9 plots the observer speeds versus actual and *J-*adjusted bird detection frequencies.

Prior to movement rate compensation, detection numbers per observer speed class decreased significantly with increasing observer speed (fantail: Pearson *r = −*0.79, *p* = 0.006, *n* = 10; wattlebird: *r* = *−*0.71, *p =* 0.004, *n* = 14). After compensation, neither correlation was statistically significant (fantail: *r* = *−*0.002, *p* = 0.99, *n* = 10; wattlebird: *r* = *−*0.361, *p* = 0.161, *n* = 14), suggesting that the movement compensation procedure was effective.

### Test 3: A difficult population (low visibility, low density, high relative movement)

#### Method

*WildlifeDensity* was next applied to a scenario that is challenging to study by distance sampling—a low-density population of a small, highly mobile forest bird. We again studied the grey fantail, a small (9 g; [25]), actively mobile (*ū* = 116 m/min; unpubl. data), territorial, arboreal insectivorous species that captures much of its food on the wing. The population was sampled within a small floodplain containing open eucalypt forest in the Otway Ranges, Victoria, Australia. The area is bounded by a creek on one side and a hillside on the other, forming a flat, well-defined 10-ha area. Two roughly parallel transects, each 600 m in length, were walked in consistent weather conditions several times per month in the October–December breeding seasons [23] of 2016–2019 using the survey protocol (Section *A survey protocol*). Detections per survey day were few (0–16), so the data were combined into monthly sets, which is reasonable as the survey conditions and observer were consistent. Even so, numbers were still quite low, with only 5–44 detections per month (mean = 21.6/mo). Densities were again estimated using the methods of Test 1.

Because the true population densities were unknown, they were approximated by the best estimates obtainable. These were based on intensive observations of fantail nesting, their nests recognisable by their distinctive “wine glass” shape. All fantails observed at the site during the breeding season were found to be associated with nests in pairs. Consequently, detailed searches and mapping were conducted to locate all active nests (those with recently laid eggs, brooding fantails or fledglings), with the number of fantails assumed to be double the number of nests. The numbers of nesting pairs in the 10-ha site were estimated as two in 2016, three in both 2017 and 2018, and zero in 2019, giving fantail densities of 0.4, 0.6, 0.6 and 0.0 per ha, respectively. We used these densities as “best estimates” of the true densities. We consider them informative rather than definitive; a better demonstration could be made when a suitable dataset becomes available.

We assumed that each observation of incubation activity was associated with one of the breeding pair being largely undetectable (i.e., stationary on the nest). The numbers undetectable were divided by the likely total number of birds (number detected on survey plus number undetectable) to estimate the proportions of the population readily observable in each month and breeding season. These ranged between 0.43 and 1 for the monthly estimates, and were 0.98, 0.72, 0.91 and 1.00 for breeding seasons of 2016, 2017, 2018 and 2019, respectively. These proportions were entered in the corresponding *WildlifeDensity* and *Distance* analyses using the options *proportion observable* and *sampling fraction*, respectively, which increased both types of density estimates to similar extents.

#### Results

Shapiro-Wilk tests showed that *WildlifeDensity’s* monthly density estimates (*n* = 12) had an approximately normal (Gaussian) distribution (mean = 0.255 SD = 0.098, *W* = 0.949, *p* = 0.615 for radial data; mean = 0.266, SD = 0.158, *W* = 0.926, *p* = 0.336 for perpendicular), whereas *Distance* estimates were only marginally so (mean = 0.988, SD = 0.623, *W* = 0.864, *p* = 0.055). A related-samples Friedman’s two-way analysis of variance by ranks was therefore used to test the hypothesis that the three distributions were the same. No significant difference was found between the densities estimated by *WildlifeDensity* using radial and perpendicular data (Friedman statistic = 0.167, *p* = 0.683); however, both radial and perpendicular estimates were significantly less than those estimated by conventional *Distance* (Friedman statistics = −1.583 and −1.417, respectively, with both *p* < 0.001).

**Fig 10:**
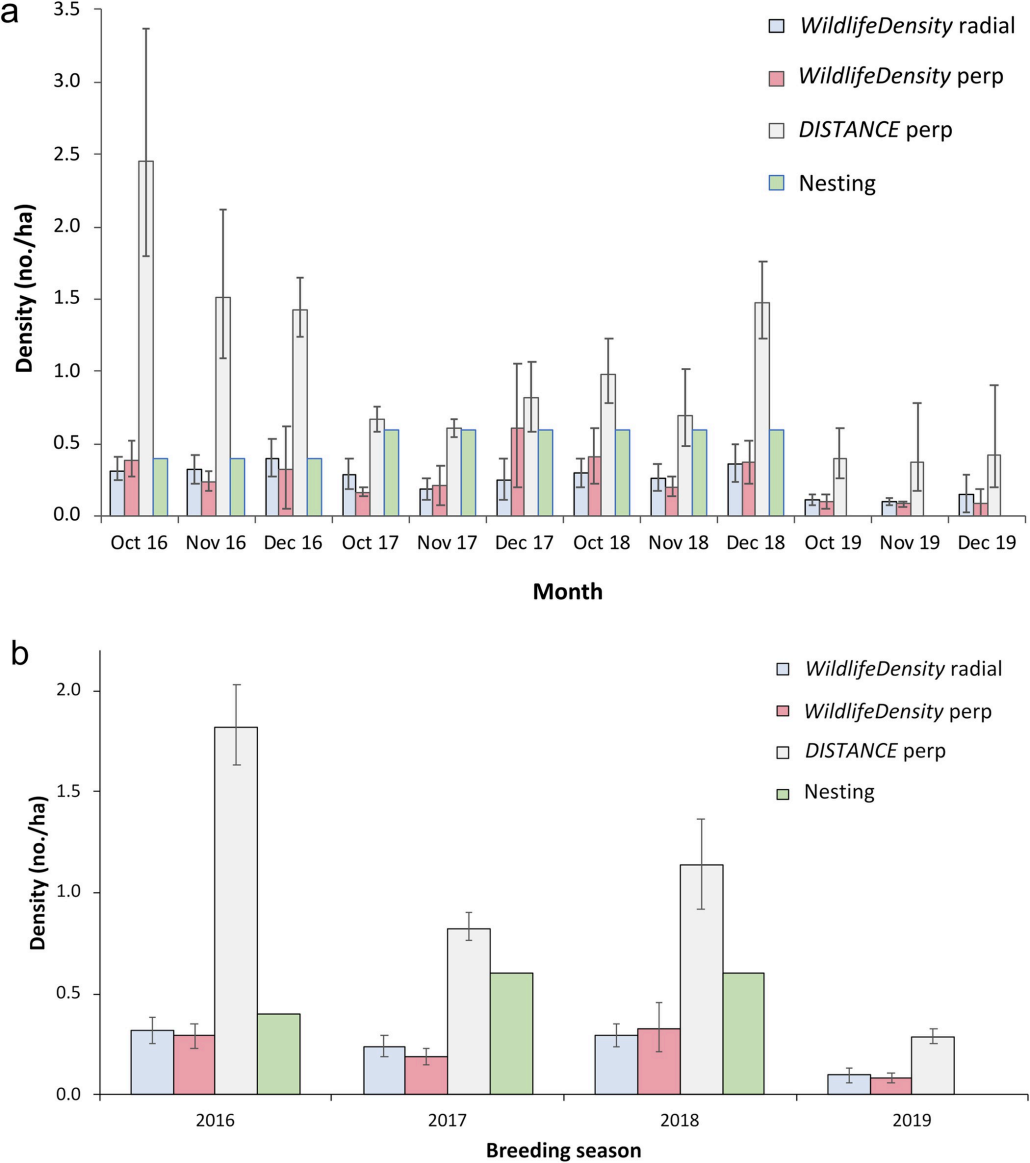
Comparison of grey fantail densities (with 95% confidence intervals) estimated by four methods. Data are pooled a) monthly and b) for October–December breeding seasons. No nests were completed in 2019.

With the nesting densities of grey fantails in the survey area known approximately (0.4, 0.6, 0.6 and 0.0 per ha in 2016, 2017, 2018 and 2019, respectively), and the *Distance* estimates showing only marginal normality, the nonparametric Wilcoxon signed-ranks test was then used to compare the fantail nesting densities with the estimates from the three line transect methods. The nesting results showed fairly large but non-significant differences from the *WildlifeDensity* radial estimates (*n* = 12, *W* = 12 (12,54), *p* = 0.067, effect size *r* = 0.6, skewness = –0.22) and perpendicular estimates (*n =* 12, *Z* = 1.8, *W =* 16 (16,62), *p =* 0.077, *r =* 0.5, skewness = 0.32). In contrast, *Distance* estimates showed large differences that were statistically significant (*n =* 12, *W* = 0 (78,0), *p <* 0.001, *r =* –1.0). The results are shown graphically for the twelve nesting months (Fig 10A) and after pooling across each nesting season (Fig 10B). The fit of the two WD estimators was judged to be acceptably close, especially in view of the variability in the contributary data (e.g., detection distances, fantail and observer movement speeds), the low samples sizes and the semi-closed structure of the habitat.

## Discussion

The three tests demonstrate that *WildlifeDensity* can use distance sampling data to estimate wildlife population density with acceptable accuracy. On the benchmark datasets (Test 1), *WildlifeDensity* estimates were similar to those of *Distance*, suggesting that both approaches are based on realistic models. However, the assumptions required for these models differ somewhat.

### Measurement accuracy

With the benchmark datasets, the estimates produced by *WildlifeDensity* using radial distances were at least as good as those of *WildlifeDensity* and *Distance* using perpendicular distances. Radial distances are preferred as they have a number of advantages; primarily, being simple and accurate to measure using a laser rangefinder. Perpendicular distances, meanwhile, require measurements of radial distance *and* lateral angle, the latter typically being measured by sight using a protractor and/or compass in relation to the transect line, which is usually unmarked. This increases the observer’s workload and the potential for measurement error [26], particularly when animals are moving and/or numerous. Also, use of the search protocol (Section *A survey protocol*) is intended to reduce error by promoting uniform observer behaviour in scanning and measurement. Surveys conducted this way may also be analysed in *Distance* if lateral angles are recorded. Conversely, data collected for *Distance* purposes may be analysed in *WildlifeDensity* only if the search protocols are consistent, noting that a *Distance* protocol may bias scanning the area directly ahead to avoid missing detections on the transect line, with other search behaviours largely left up to the researcher [2].

### Detection certainty on the transect line

Previous distance sampling approaches have found it difficult to model radial data, with the result that perpendicular models have prevailed [6]. These require a detection function that peaks at zero distance (i.e., at the transect), where detectability must be assumed to be complete. This is a problematic assumption, as detectability is more closely related to the direct-line distance from the observer, not the transect line. Numerous techniques for compensating for incomplete detection on the transect have been devised for use with the *Distance* method, such as double-observer methods [27]. Furthermore, detections near the transect line, where perpendicular distances are short and radial distances are often relatively high, can be particularly prone to measurement error, distorting the detection function and causing bias [28]. *WildlifeDensity* overcomes these issues by modelling direct-line detection probability based on system geometry, animal conspicuousness and visual attenuation.

### Movement

Conventional *Distance* is a “snapshot” method based on an assumption that animals do not move. In reality, animals do move, although many move slowly enough to have little effect on density estimates (particularly when the animal’s speed is less than half the observer’s [29]). Those that are highly mobile (many birds) may produce density overestimates [13], thereby preventing such animals from being accurately surveyed by distance sampling. The data collected in Test 3 show the scale of density overestimates that can result when movement is not considered. The fantail detection rate, represented by the value of the movement correction factor *J*, varied between 3.5 and 6.4 times the numbers expected if the birds were immobile, which is consistent with the differences between the *WildlifeDensity* and *Distance* estimates.

The relative movement problem has prompted modified distance sampling techniques for specific situations. For example, seabird surveys may divide sea-surface line transects into a series of “snapshot” point surveys [30] or adjust density estimates based on vector analysis of seabird–observer relative speed and direction [31]. These techniques require the point or strip half-width to be significantly limited (typically to 300 m) so that complete detectability within the strip can be assumed, which is unrealistic for some seabirds and reduces the sample size [32].

In [13], simulations of movement effects on detectability predicted a comparable although different relationship between animal–observer relative speed and detection rate bias to that reported in the present study (Fig 8). An R software package was devised to implement it in analyses of distance sampling with movement; interestingly, it uses radial detection data [33]. This approach is computationally intensive and somewhat complex to use. In comparison, to compensate for movement, the *WildlifeDensity* application simply requires input of the population speed and transect lengths and durations. Test 2 showed how this approach can compensate quite precisely and relatively easily for the effect of animal–observer movement on detection frequency. Together, the three tests show that *WildlifeDensity* can provide a useful tool for researchers of mobile wildlife. While we have also used *WildlifeDensity* over many years in surveys of a wide variety of species and habitats, data on mobile populations of known density have always been lacking. Further study of such populations is needed to comprehensively validate *WildlifeDensity* in different scenarios.

### Fitting detection functions

The Nelder-Mead function minimization approach used in *WildlifeDensity* is a direct search simplex method that has been criticised for being unable to prove convergence mathematically [20] or, sometimes, failing due to convergence to a non-stationary point (e.g., [34]). However, failure has been shown to occur mainly where the number of dimensions is high and the simplex shape is unduly distorted during expansion and contraction [35]. The method performs well with small-dimensional real-life problems such as those solved by *WildlifeDensity*, which employs only 2–3 dimensions. We have not encountered any failures to model detection probability using this method.

### Summary

In summary, *WildlifeDensity* can use radial distances, does not require complete detectability on the transect, and compensates for animal–observer movement. *WildlifeDensity* also has other applications not discussed here due to space limitations. For example, its modelling of direct-line distances allows more accurate estimates in three-dimensional scenarios (e.g., flying birds, tall forests), whereas conventional techniques model distances as if on a horizontal plane. In *WildlifeDensity*, the mean population elevation difference can be entered to compensate for differences between direct-line and horizontal radial distances. It can also be used in fixed-point and aerial surveys, with observer altitude and speed entered into the program. When repeated surveys of a site are required, such as for population monitoring, these can be performed without distance measurement if the detectability (detectability coefficient *S*) is known and assumed to be temporally constant. This further simplifies fieldwork by making animal counts the only necessary data. These benefits expand the potential applications of distance sampling, making *WildlifeDensity* a useful conservation tool.

## References

[1] Fryxell JM, Sinclair AR, Caughley G. Wildlife ecology, conservation, and management. John Wiley & Sons; 2014.

[2] Buckland ST, Rexstad EA, Marques TA, Oedekoven CS. Distance sampling: methods and applications. New York: Springer; 2015.

[3] Thomas L, Buckland ST, Burnham KP, Anderson DR, Laake JL, Borches DL, et al. Distance sampling. In El-Sharawi AH & Piegorsch WW, editors. Encyclopaedia of environmetrics. UK: John Wiley & Sons; 2002. pp. 544–552.

[4] Buckland ST, Anderson DR, Burnham KP, Laake JL. Distance sampling. London, UK: Chapman and Hall; 1993.

[5] Thomas L, Buckland ST, Rexstad EA, Laake JL, Strindberg S, Hedley SL, et al. Distance software: design and analysis of distance sampling surveys for estimating population size. J Appl Ecol. 2010;47(1): 5–14. doi: 10.1111/j.1365-2664.2009.01737.x 20383262 PMC2847204

[6] Buckland ST, Plumptre AJ, Thomas L, Rexstad EA. Line transect sampling of primates: can animal-to-observer distance methods work? Int J Primatol. 2010;31: 485–99.

[7] Glass R, Forsyth DM, Coulson G, Festa-Bianchet M. Precision, accuracy and bias of walked line-transect distance sampling to estimate eastern grey kangaroo population size. Wildl Res. 2015;42(8): 633–41.

[8] Southwell C. Evaluation of walked line transect counts for estimating macropod density. J Wildl Manage. 1994: 348–56.

[9] Chen SX, Cowling A. Measurement errors in line transect surveys where detectability varies with distance and size. Biometrics. 2001;57(3): 732–42. doi: 10.1111/j.0006-341x.2001.00732.x 11550922

[10] Laake JL, Borchers DL. Methods for incomplete detection at distance zero. In: Buckland ST, Anderson DR, Burnham KP, Laake JL, Borchers DL, Thomas L, editors. Advanced distance sampling: estimating abundance of biological populations. Oxford, UK: Oxford University Press; 2004.

[11] Becker EF, Christ AM. A unimodal model for double observer distance sampling surveys. PLoS One. 2015;10(8): e0136403. doi: 10.1371/journal.pone.0136403 26317984 PMC4552872

[12] Buckland ST, Anderson DR, Burnham KP, Laake JL, Borchers DL, Thomas L. Introduction to distance sampling: estimating abundance of biological populations. Oxford University Press; 2001.

[13] Glennie R, Buckland ST, Thomas L. The effect of animal movement on line transect estimates of abundance. PloS One. 2015;10(3): e0121333. doi: 10.1371/journal.pone.0121333 25799206 PMC4370374

[14] University of Melbourne. WildlifeDensity (version 2.5) [Computer software] Parkville, Australia: University of Melbourne; 2018. Available from: https://biosciences.unimelb.edu.au/research/facilities-equipment-and-resources

[15] Morgan DG, Pegler P. 30 Managing a kangaroo population by culling to simulate predation: the Wyperfeld trial. In Coulson GM, editor. Macropods: the biology of kangaroos, wallabies, and rat-kangaroos. Collingwood, VIC, Australia: CSIRO Publishing; 2010. pp. 349–360.

[16] Alldredge MW, Simons TR, Pollock KH. A field evaluation of distance measurement error in auditory avian point count surveys. J Wildl Manage. 2007;71(8): 2759–66.

[17] Yapp WB. The theory of line transects. Bird study. 1956;3(2): 93–104.

[18] Skellam JG. The mathematical foundations underlying the use of line transects in animal ecology. Biometrics. 1958;14(3): 385–400.

[19] Nelder JA, Mead R. A simplex method for function minimization. Comput J. 1965;7(4): 308–13.

[20] Wright MH. Nelder, Mead, and the other simplex method. Doc Math. 2010;7: 271–6.

[21] Shaw DE, Wedderburn RWM. MINIM: Minimisation of mathematical functions. CSIRO Division of Mathematical Statistics.

[22] Thompson SK. Stratified sampling. In: Thompson SK, editor. Sampling. 3rd ed. John Wiley & Sons; 2012. pp. 141–156.

[23] Burnham KP, Anderson DR, Laake JL. Estimation of density from line transect sampling of biological populations. Wildlife monographs. 1980(72): 3–202.

[24] Hone J. A test of the accuracy of line and strip transect estimators in aerial survey. Wildl Res. 1988;15(5): 493–7.

[25] Munro K. Breeding behaviour and ecology of the grey fantail (Rhipidura albiscapa). Aust J Zool. 2007;55(4): 257–65.

[26] Chen SX. Measurement errors in line transect surveys. Biometrics. 1998: 899–908. 10.1111/j.0006-341x.2001.00732.x 11550922

[27] Buckland ST, Turnock BJ. A robust line transect method. Biometrics. 1992: 901–9.

[28] Marques TA. Predicting and correcting bias caused by measurement error in line transect sampling using multiplicative error models. Biometrics. 2004;60(3): 757–63. doi: 10.1111/j.0006-341X.2004.00226.x 15339299

[29] Hiby AR. The effect of random whale movement on density estimates obtained from whale sighting surveys. Rep Int Whaling Commission. 1982;32: 791–3.

[30] Tasker ML, Jones PH, Dixon TI, Blake BF. Counting seabirds at sea from ships: a review of methods employed and a suggestion for a standardized approach. Auk. 1984;101(3): 567–77.

[31] Spear L, Nur N, Ainley DG. Estimating absolute densities of flying seabirds using analyses of relative movement. Auk. 1992;109(2): 385–9.

[32] Barbraud C, Thiebot JB. On the importance of estimating detection probabilities from at‐sea surveys of flying seabirds. J Avian Biol. 2009;40(6): 584–90.

[33] Glennie R, Buckland ST, Langrock R, Gerrodette T, Ballance LT, Chivers SJ, et al. Incorporating animal movement into distance sampling. J Am Stat Assoc. 2021;116(533): 107–15.

[34] Powell MJ. Direct search algorithms for optimization calculations. Acta numer. 1998;7: 287–336.

[35] Gao F, Han L. Implementing the Nelder-Mead simplex algorithm with adaptive parameters. Comput Optim Appl. 2012;51(1): 259–77.

